# Supplementary material for: Direct single-molecule detection and super-resolution imaging with a low-cost portable smartphone-based microscope
Source: Nat Commun. 2025 Oct 8;16:8937. doi: 10.1038/s41467-025-63993-z (PMC12508040; doi:10.1038/s41467-025-63993-z)
Supplement: Supplementary file 1 — Supplementary Information [file 41467_2025_63993_MOESM1_ESM.pdf]

Supplementary Information for

Direct single-molecule detection and super-resolution imaging with a low-cost portable smartphone-based microscope

Morgane Loretan[1], Mariano Barella[1,2,], Nathan Fuchs[1], Samet Kocabey[2,3], Karol Kołątaj[1,2], Fernando D. Stefani[4,*], Guillermo P. Acuna[1,2,*]*

1. Department of Physics, Faculty of Science and Medicine, University of Fribourg, University of Fribourg, Chemin du Musée 3, PER 08, Fribourg, CH-1700, Switzerland
2. Swiss National Center for Competence in Research (NCCR) Bio-inspired Materials, University of Fribourg, Chemin des Verdiers 4, CH-1700 Fribourg, Switzerland
3. Department of Oncology, Microbiology and Immunology, Faculty of Science and Medicine, University of Fribourg, Chemin du Musée 18, PER 17, Fribourg, CH-1700, Switzerland
4. Centro de Investigaciones en Bionanociencias (CIBION), Consejo Nacional de Investigaciones Científicas y Técnicas (CONICET), Godoy Cruz 2390 (C1425FQD) and Departamento de Física, Facultad de Ciencias Exactas y Naturales, Universidad de Buenos Aires, Güiraldes 2620 (C1428EHA), Ciudad Autónoma de Buenos Aires, Argentina

Corresponding authors:

*mariano.barella@unifr.ch

*fernando.stefani@df.uba.ar

*guillermo.acuna@unifr.ch

Table of Contents

Supplementary Note 1. Smartphone-based setup configurations 3

Supplementary Note 2. Components of the smartphone-based setup 4

Supplementary Note 3. TEM of the 2LS, monomer and dimer 8HB DNA origami nanostructures..... 5

Supplementary Note 4. Single ATTO 647N intensity traces in the high-end microscope 7

Supplementary Note 5. Characterization of dye incorporation in the 2LS DNA origami nanostructures 8

Supplementary Note 6. Direct single-molecule detection with more smartphones 10

Supplementary Note 7. SBR and SNR of ATTO 542 molecules in a high-end microscope................ 12

Supplementary Note 8. Specifications of the smartphone-based microscope 13

Supplementary Note 9. Representative drift trajectories of the microscopes................................ 15

Supplementary Note 10. Localization precision estimation for DNA-PAINT with the smartphone-based microscope 16

Supplementary Note 11. Kinetics and optimization of DNA-PAINT experiments............................ 18

Supplementary Note 12. Example of a super-resolved microtubule network 21

Supplementary Note 13. Resolution estimation 22

Supplementary Note 14. TIR excitation design simulations 23

Supplementary Note 15. Super-resolution on an office desk 24

Supplementary Note 16. High-end widefield fluorescence microscope 25

Supplementary Note 17. 2LS DNA origami staples list 26

Supplementary Note 18. 8HB DNA origami staples list 33

Supplementary Note 19. Experimental parameters 42

Supplementary References.. 46

Supplementary Note 1. Smartphone-based setup configurations

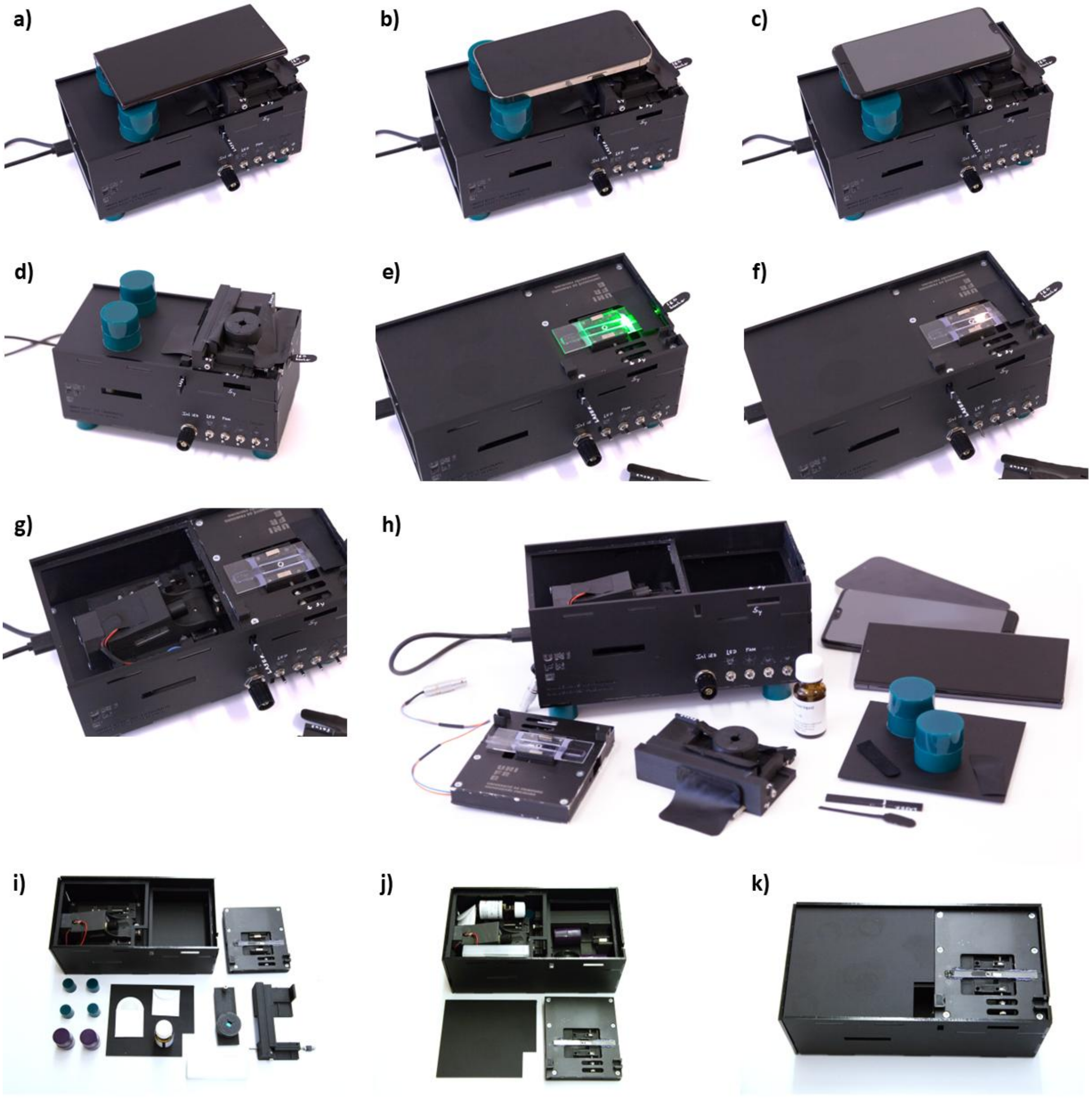

Supplementary Figure 1: Smartphone-based setup configurations. *Different configurations of the smartphone-based setup featuring (a) a Samsung Galaxy S22 Ultra, (b) an iPhone 14 Pro, (c) a Huawei P20 Pro, and (d) no smartphone. (e) Top view without the objective stage. Fluorescence mode, laser on. (f) Brightfield mode, white LED on. (g) Half-cover removed. The laser stage and sample stage are visible. (h) Smartphone-based microscope modules, immersion oil, beam blockers, slip-resistant feet, and the smartphones used in this work. (i) and (j) Packing the smartphone-based microscope for transportation (initial version of the setup). (k) Microscope ready for transportation.*

Supplementary Note 2. Components of the smartphone-based setup

Supplementary Table 1 lists the components of the smartphone-based setup and their approximate prices in Switzerland (as of November 2022).

***Supplementary Table 1**: Price list of relevant components (production costs were excluded).*

**Component**	**Price (€)**
Objective (B07FXVJVDP, Richer-R)	11.00
Half-ball lens (#45-934, Edmund)	45.00
Emission filter (10CGA-550, Newport)	57.75
Optical glue, a fraction (NOA65, Thorlabs)	2.00
Laser + heatsink (CW532F-020F, Roithner LaserTechnik)	70.35
Electronics (connectors, cables, potentiometer, switch, fan, LED, voltage regulator)	26.30
Battery (EB-P3300, Samsung)	53.55
Silicone feet (Ecoflex 00-50, Smooth-on)	4.83
Frame (3D-printed parts, laser-cut parts, glue)	29.13
Miscellaneous (screws, knobs, bolts, threaded insert, magnets)	33.76
Smartphone app	13.00
**Total**	346.67

Apps. We used the following camera apps for recording in RAW mode: MotionCam Pro, RAW Video app version 2.0.2-pro (Samsung Galaxy S22 Ultra, ~ 13 €); Camera Pro by Moment (Apple iPhone 14 Pro, ~ 9 €); FreeDCam (Huawei P20 Pro, free, open-source).

Supplementary Note 3. TEM of the 2LS, monomer and dimer 8HB DNA origami nanostructures

For TEM imaging, 5 µl of sample solution were dropped onto formvar-coated grids (300 mesh Cu, 22-1MHC30-50, Micro to Nano). After 1 min, the solution was removed with a paper filter, and the sample was negatively stained for 10 s using 2% Uranyl acetate solution. TEM images were acquired with a FEI Tecnai Spirit microscope with an accelerating voltage of 120 kV.

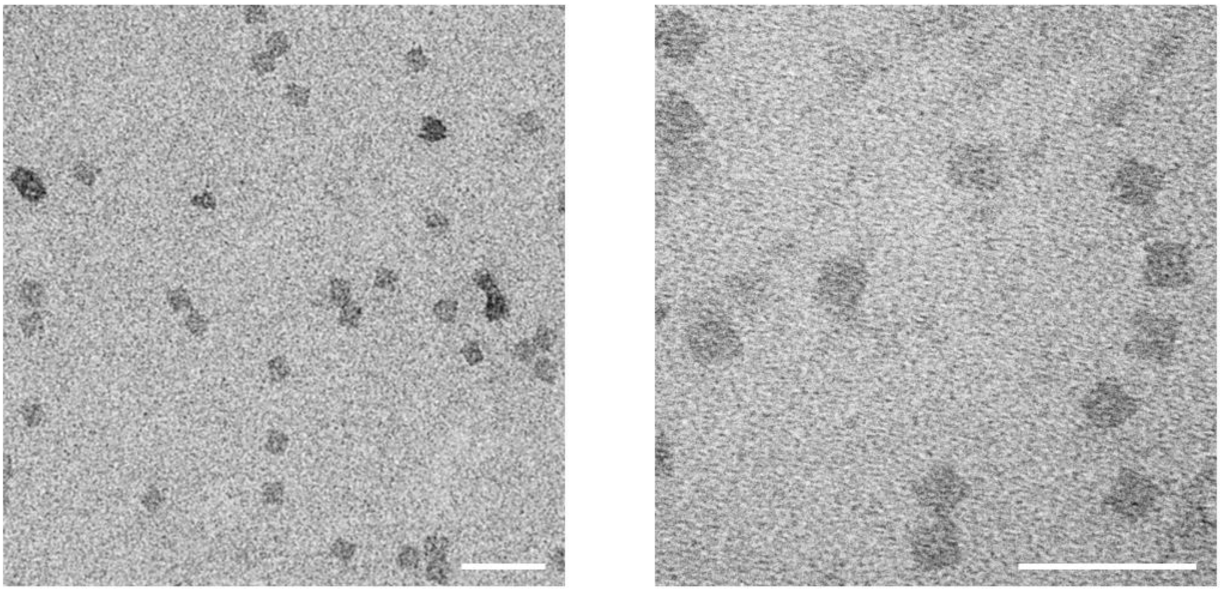

Supplementary Figure 2: TEM images of purified 2LS nanostructures. *Scale bars: 200 nm.*

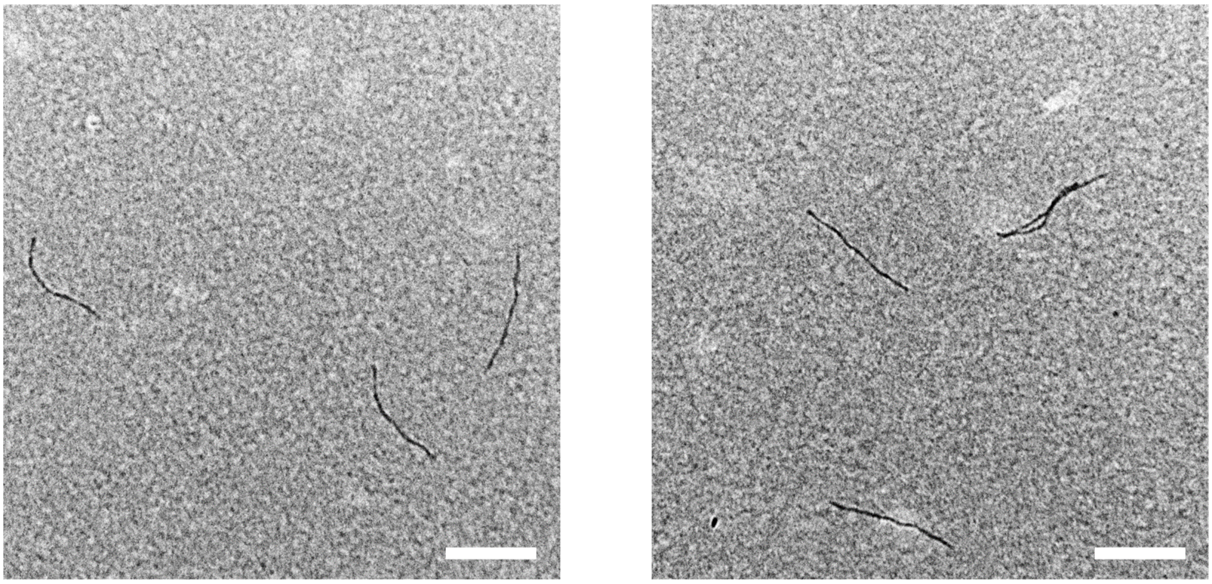

Supplementary Figure 3: TEM images of purified monomer 8HB nanostructures. *Scale bars: 200 nm.*

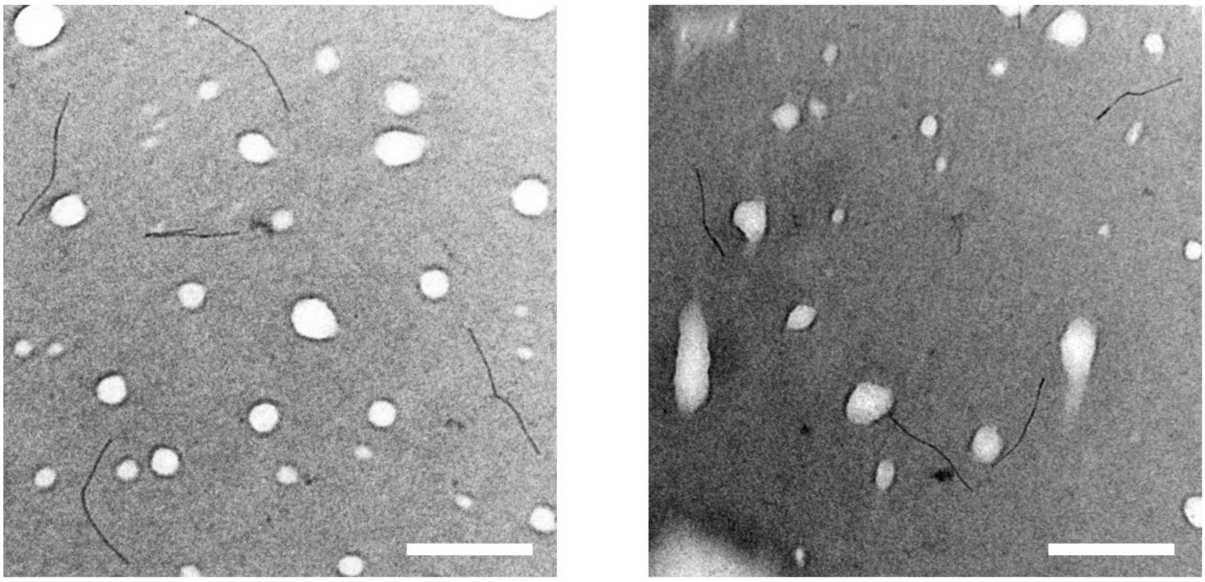

Supplementary Figure 4: TEM images of purified dimer 8HB nanostructures. *Scale bars: 500 nm.*

Supplementary Note 4. Single ATTO 647N intensity traces in the high-end microscope

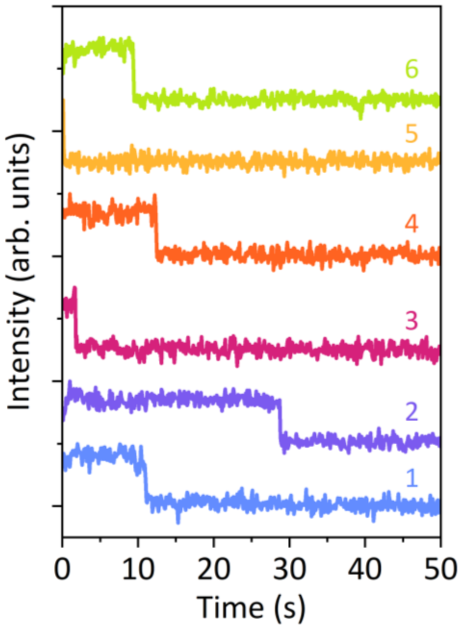

Supplementary Figure 5: Single-step intensity traces of ATTO 647N single molecules acquired with the high-end microscope. *The numbers indicate the 2LS nanostructure of Figures 2d and 2f. The level of the signal after photobleaching corresponds to the background level. Source data are provided as a Source Data file.*

Supplementary Note 5. Characterization of dye incorporation in the 2LS DNA origami nanostructures

To characterize the percentage of 2LS origami nanostructures containing both dyes (ATTO 542 and ATTO 647N) and those having only one, the samples were studied using the high-end microscope and 1×PPC imaging buffer. First, the nanostructures were excited with a 640 nm laser until the red fluorophores were photobleached, and, subsequently, the same region of interest was excited with a 532 nm laser until the green ones were photobleached too. Intensity traces of fluorescent spots were analyzed. Traces showing only single steps were considered. All other cases were discarded, e.g., two steps, blinking, or spots that didn't photobleach. After counting single molecules that spatially colocalize, we found that 87% of the nanostructures had single ATTO 542 molecules (Supplementary Figure 6a), 85% had single ATTO 647N molecules (Supplementary Figure 6b), and 72% had incorporated both dyes (Supplementary Figure 6c). This result is in agreement with what is expected from the incorporation yield of dyes into a DNA origami, plus some possible contribution from photobleaching of the dyes. For dye incorporation, we followed a reported protocol that can reach 82% efficiency for single dyes[1]. If such a maximum yield is reached for both dyes, it is expected that 97% of the origamis have at least one of the two dyes and that 67% of the origamis have both fluorophores. Our observations retrieve a comparable value for the fraction of doubly labeled origami. In Supplementary Figure 6d, colocalization between the smartphone-based and high-end microscopes is shown. Due to the PSF size of the smartphone-based setup, 1:1 and 2:1 cases were taken into account. The data correspond to 41 fluorescent events registered in Figure 2f of the main text, out of which 87% were considered to come from DNA origami nanostructures.

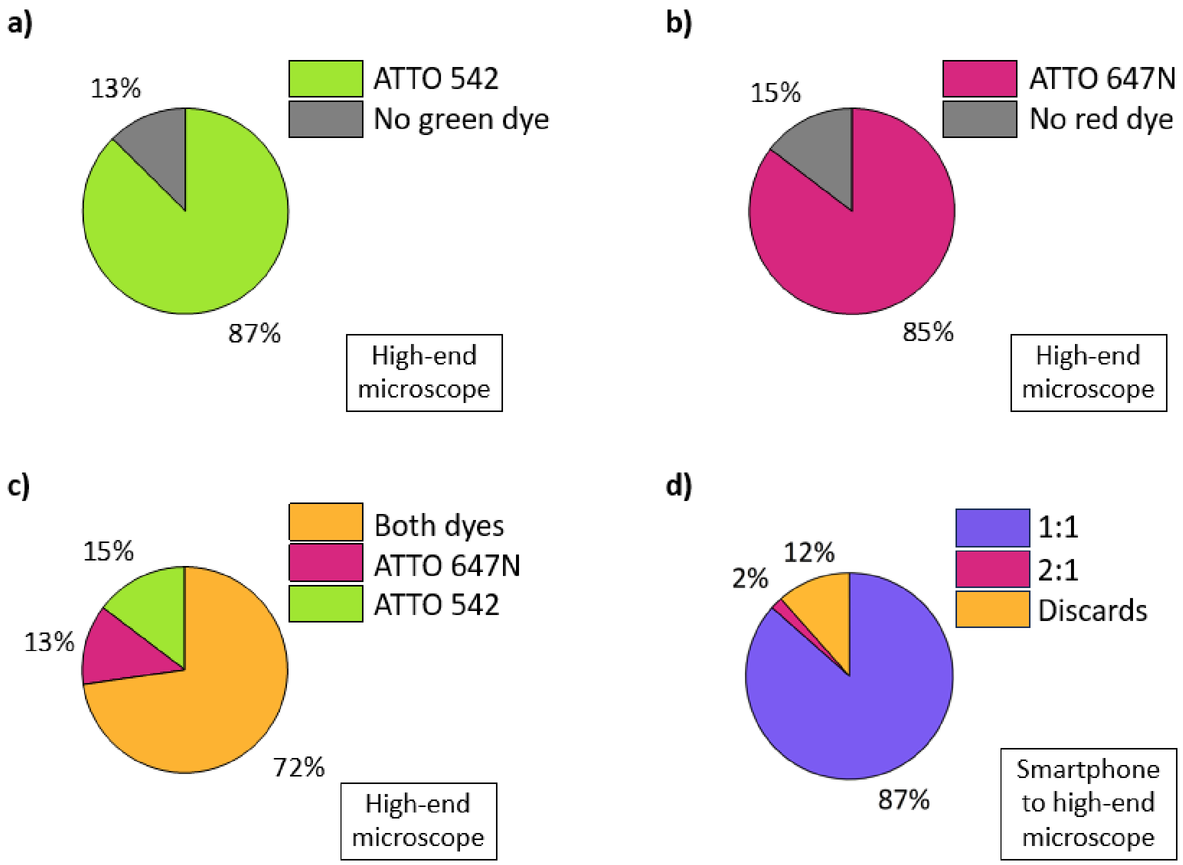

Supplementary Figure 6: Single-molecule incorporation in 2LS using the high-end microscope. *Percentage of nanostructures showing a single ATTO 542 (a), a single ATTO 647N (b), and both molecules (c). A total of 143 nanostructures were analyzed, of which 104 presented both dyes, 122 presented single ATTO 647N molecules, and 125 presented single ATTO 542 molecules. (d) Colocalization between microscopes. Legend N:M represents the number of ATTO 542 photobleaching steps observed in the smartphone-based setup (N) against the number of ATTO 647N photobleaching steps observed in the high-end microscope (M). Source data are provided as a Source Data file.*

Supplementary Note 6. Direct single-molecule detection with more smartphones

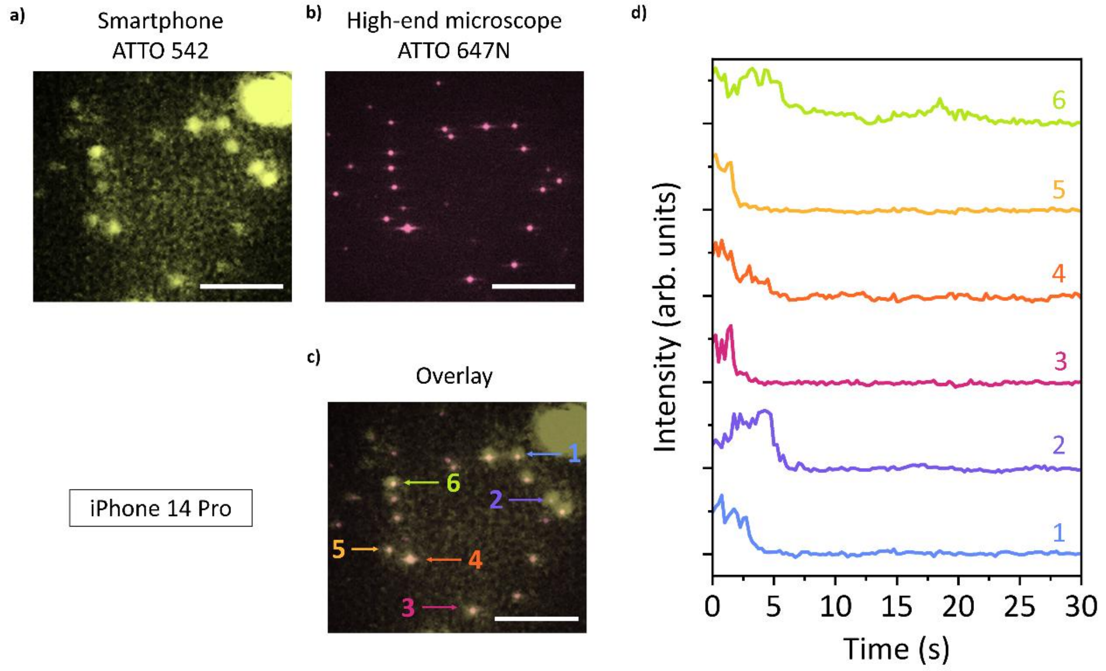

Supplementary Figure 7: Direct single-molecule detection with the smartphone-based microscope using an iPhone 14 Pro. *(a) Fluorescence image of the 2LS origami on quartz, detecting only the ATTO 542 with the smartphone-based microscope and (b) the ATTO 647N with the high-end microscope. (c) Overlay of images (a) and (b). Arrows and numbers indicate intensity traces plotted in (d). (d) ATTO 542 intensity traces vs. time. They correspond with the 2LS nanostructures indicated in the overlayed images shown in (c). Scale bars: 10 µm. Source data are provided as a Source Data file.*

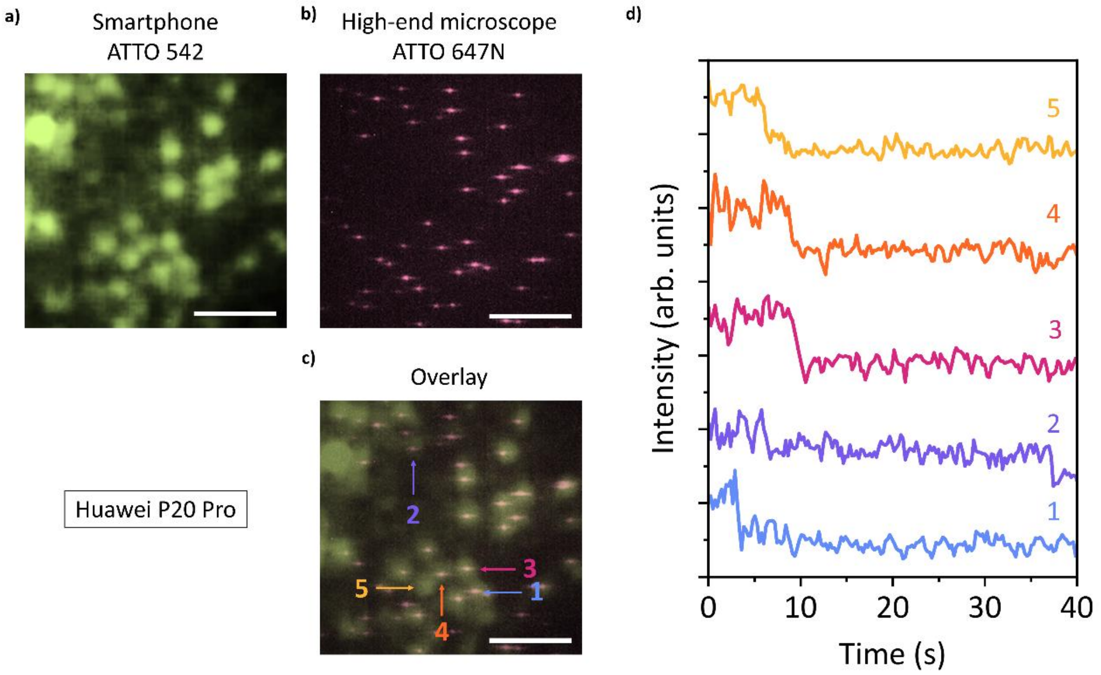

Supplementary Figure 8: Direct single-molecule detection with the smartphone-based microscope using a Huawei P20 Pro. *(a) Fluorescence image of the 2LS origami on quartz, detecting only the ATTO 542 with the smartphone-based microscope and (b) the ATTO 647N with the high-end microscope. (c) Overlay of images (a) and (b). Arrows and numbers indicate intensity traces plotted in (d). (d) ATTO 542 intensity traces vs. time. They correspond with the 2LS nanostructures indicated in the overlayed images shown in (c). Scale bars: 10 µm. Source data are provided as a Source Data file.*

Supplementary Note 7. SBR and SNR of ATTO 542 molecules in a high-end microscope

To compare the performance of the smartphone-based microscope with a high-end microscope, we recorded the fluorescence intensity transients of several ATTO 542 molecules deposited onto glass and quartz slides using the high-end microscope described in the Methods section. The fluorophores, incorporated in the 8HB origami monomers, were irradiated until they photobleached using similar experimental conditions as the ones used for the smartphone-based microscope measurement (see Supplementary Note 19). The exposure time was 100 ms.

Supplementary Figures 9a and 9b show the distribution of the SBR and the SNR, respectively, for 114 single molecules on glass and for 124 molecules on quartz registered under the high-end microscope. The median values of the distributions (black dots) are SBR = 0.67 (2.25) and SNR = 9.9 (16.8) for the measurements on quartz (glass). The SNR was calculated as described in the Methods section. The SBR was also calculated as described in the Methods section but using an inner ROI of 3 × 3 px^2 and an outer ROI of 10 × 10 px^2 , centered at the same position, to compensate for the pixel size difference between the two setups. To make a fair comparison and match the effective exposure time of 1.25 s we used with the smartphone-based microscope, we estimated the SBR from a 12-frame averaged image (1.2 s effective exposure time). In addition, we only considered the molecules that were constantly emitting during those 12 frames; in other words, blinking molecules were not taken into account.

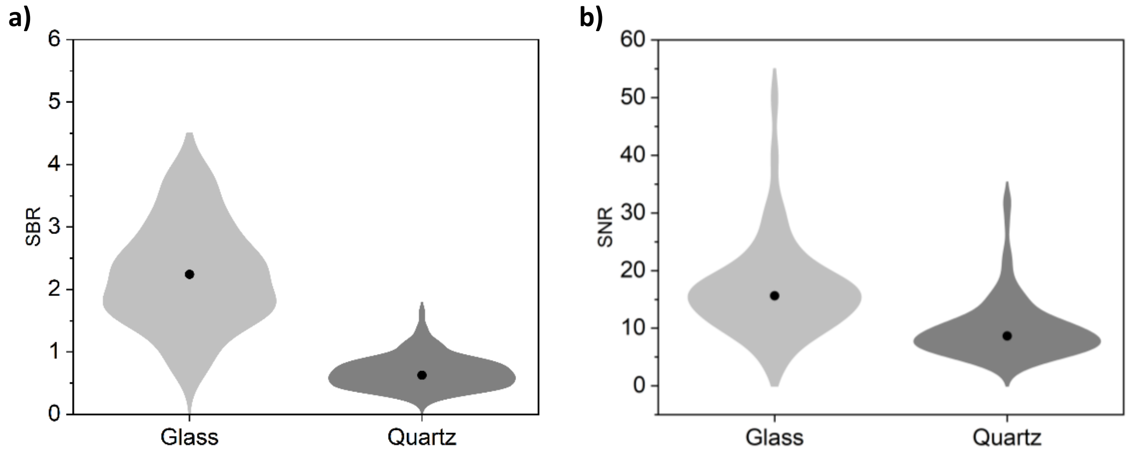

Supplementary Figure 9: SBR and SNR of ATTO 542 molecules in a high-end microscope. *Distribution of (a) SBR, or Weber contrast, and (b) SNR for 114 (124) single ATTO 542 molecules measured in the high-end microscope on glass (quartz). See Supplementary Table 13 for further details on the experimental parameters. Source data are provided as a Source Data file.*

Supplementary Note 8. Specifications of the smartphone-based microscope

Some relevant parameters of the smartphone-based microscope are reported in Supplementary Table 2. The specifications depend on the cameras that were used. The numerical aperture (NA) was obtained using the f-number (*f/#*) informed by the smartphone manufacturer, following the relationship *f/#* = 1/2NA. The theoretical PSF size was calculated as 0.61λ/NA using the maximum emission wavelength of Cy3B, λ = 570 nm. Considering Rayleigh's criterion, the same value was assigned to the theoretical resolution. To get the experimental (measured) PSF size, we fitted N = 9811 single-molecule fluorescent spots of a DNA-PAINT video with a 2D Gaussian function using Maximum Likelihood Estimation. This was done using the Localize module of Picasso software as in any standard pre-processing step. Then, PSF size is defined here as the radius of the spot where the intensity profile is $1/e^2$ of its peak intensity. This parameter is also known as the waist ω and is related to the Gaussian function standard deviation σ as $\omega = 2\sigma$. Finally, the reported PSF size ω as the average waist $\omega = \frac{1}{N}\sum_{\mathrm{i}=1}^{N}\omega_i$, where ω_i is the average waist of the *x*-axis and *y*-axis waists, $\omega_i = \frac{\omega_{i,x}+\omega_{i,y}}{2}$, of the i-th molecule.

***Supplementary Table 2**: Specifications of the smartphone-based microscope according to the smartphone's camera.*

<table>
<tr><th colspan="4">Smartphone-based microscope specifications</th></tr>
<tr><td>Weight (kg)</td><td colspan="3">1.2</td></tr>
<tr><td>Dimensions</td><td colspan="3">11 cm × 22 cm × 12 cm</td></tr>
<tr><td>Travel range</td><td colspan="3">Laser stage: x = 22 mm / y = 10 mm / z = 10 mm
Sample stage: x = 15 mm / y = 7.5 mm
Objective stage: x = 16 mm / y = 17 mm / z = 8 mm</td></tr>
<tr><td>Objective lens</td><td colspan="3">1.7 mm focal length with f-number 2.0</td></tr>
<tr><td>Excitation wavelength (nm)</td><td colspan="3">532</td></tr>
<tr><td>Emission filter spectral range</td><td colspan="3">Cut-on at 550 nm (long-pass filter)</td></tr>
<tr><td>Field of Illumination
(x waist × y waist)</td><td colspan="3">45.6 µm × 27.6 µm</td></tr>
<tr><td>Power stability</td><td colspan="3">< 5% (fan OFF) / < 1% (fan ON) @ at room temperature for 2 h</td></tr>
<tr><td>Power at the sample plane (mW)</td><td colspan="3">17</td></tr>
<tr><td>Smartphone</td><td>Samsung Galaxy S22 Ultra</td><td>iPhone 14 Pro</td><td>Huawei P20 Pro</td></tr>
</table>

Camera type	Telephoto	Telephoto	Wide color
Sensor size - pixels (W × H)	3648 × 2736	-	-
Bit depth	16	16	16
TL focal length (mm)	70	77	27
f/#	*f*/2.4	*f*/2.8	*f*/1.8
Numerical aperture (NA)	0.21	0.18	0.28
Full field of view – FOV	ϕ = 340 µm (circular FOV)<br>L = 240 µm (side length of the inscribed square)		Not measured
Used sensor's fraction	2.2%	14%	Not measured
Pixel size at the sample plane (µm)	0.53	0.44	0.64
Theoretical resolution (µm)	1.67	1.93	1.24
Theoretical PSF size (µm)	1.67	1.93	1.24
Experimental PSF size (µm)	1.0 ± 0.1	Not measured	Not measured

Supplementary Note 9. Representative drift trajectories of the microscopes

Supplementary Figure 10 qualitatively compares the amount of drift observed when the smartphone-based microscope is placed on a standard office desk to the drift trajectory registered with a high-end microscope during DNA-PAINT experiments.

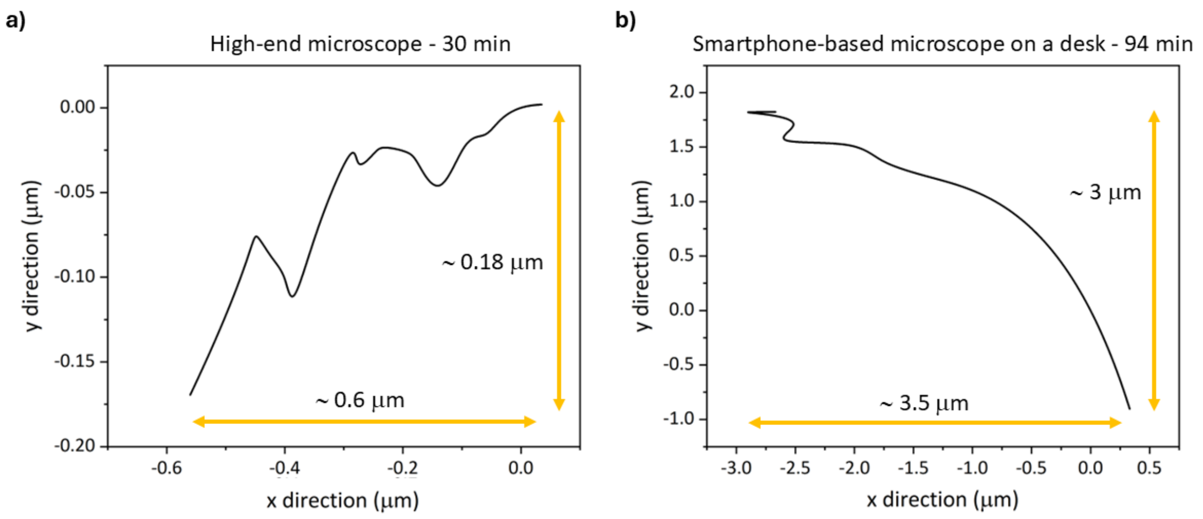

Supplementary Figure 10*: Average trajectory of the fiducial markers showing sample drift during (a) DNA-PAINT measurements performed with the high-end microscope in an optical table and (b) with the smartphone-based microscope on an office desk. Their corresponding super-resolved images are Figure 3c for (a) and Figure 4b for (b). Source data are provided as a Source Data file.*

Supplementary Note 10. Localization precision estimation for DNA-PAINT with the smartphone-based microscope

The localization precision σ reported in the main text is the standard deviation of a Gaussian fit of an ensemble of localization distributions. In detail, each localization distribution arises from several binding events of an isolated DNA origami binding site. Then, the ensemble is made by overlaying localization distributions - binding sites - using 2D cross-correlation with a pixel size of 25 nm, as indicated in the caption of Figure 3e. This step is done with Picasso's Average module and it's equivalent to aligning the binding sites by their center of mass. This method to estimate the localization precision can only be used when imaging isolated single targets such as DNA origami nanostructures and not densely labeled samples. Assuming both binding sites will have the same standard deviation, this method gives a localization precision of 84 nm, as shown in Figure 3f.

Another method, known as NeNA[2], is routinely used for the localization precision estimation when doing DNA-PAINT. Picasso's Render module incorporates this algorithm. Running NeNA on the Figure 3 dataset yields a localization precision of 119 nm, a value larger than the average standard deviation we got from the previous fittings.

A third method that uses the localization distribution of isolated binding sites and doesn't require binning for 2D cross-correlation can also be used. In detail, the localization distribution of an individual binding site is fitted with a 2D Gaussian function, and the standard deviation is retrieved. Rigorously, a covariance matrix is found for each fit, but provided there is no correlation between x and y directions, i.e., they are independent, which translates into a symmetrical localization distribution, the square root of the sum of the diagonal elements is retrieved. Then, the fitting process is applied to several nanostructures, for all their binding sites, and a distribution of standard deviation values is built. Supplementary Figure 11 shows the standard deviation distribution for the dataset of Figure 3. The median gives an estimation of 99 nm for the localization precision.

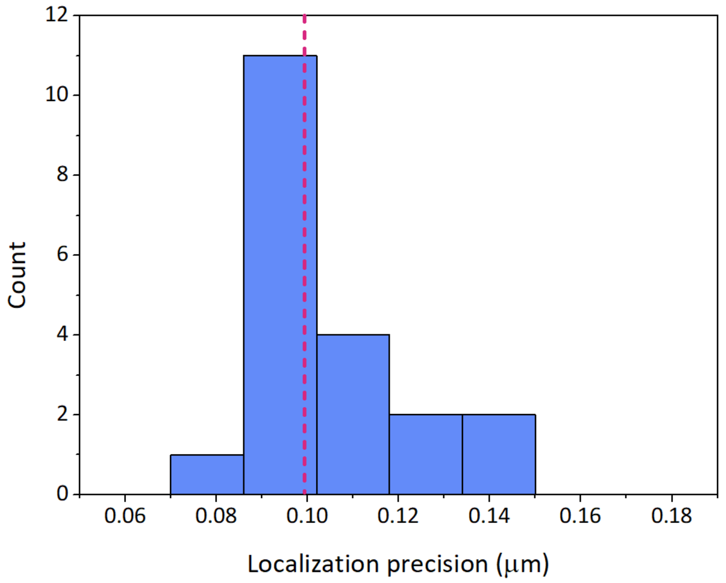

***Supplementary Figure 11**: Distribution of standard deviation values obtained from fitting 20 binding sites corresponding to the dataset of 10 8HB nanostructures presented in Figure 3. Source data are provided as a Source Data file.*

Supplementary Note 11. Kinetics and optimization of DNA-PAINT experiments

Binding events follow a mono-exponential distribution (Supplementary Figure 12) at fixed room temperature (21 °C). Data was fitted assuming a mono-exponential probability density function $pdf(t) = \left(\frac{1}{\tau}\right) e^{-t/\tau}$ in a Maximum Likelihood Estimation problem. We found an estimated mean binding time τ of (1.05 ± 0.04) s. A custom-made Python-based code was used (available at https://github.com/marianobarella/DNA-PAINT_thermometry).

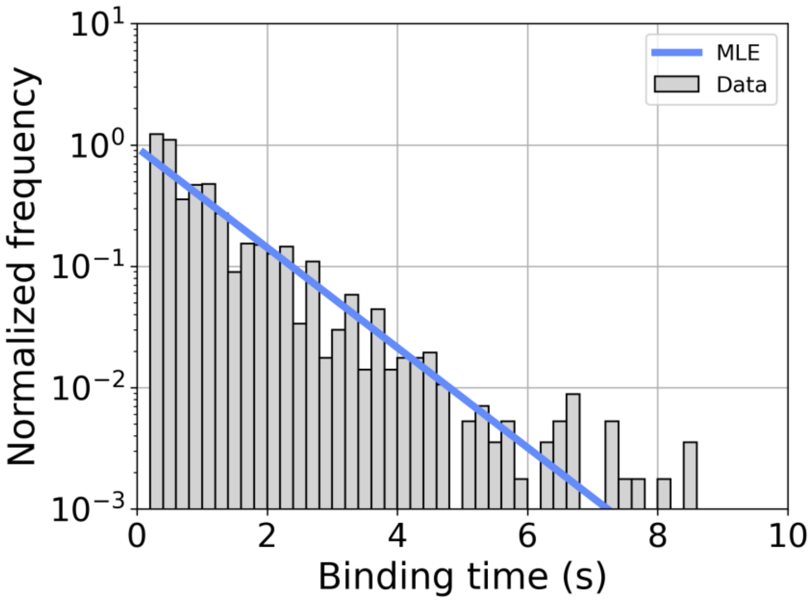

Supplementary Figure 12: Binding times distribution in DNA-PAINT kinetics. *Normalized frequency of 2836 DNA-PAINT binding events of the different binding times. The blue line represents a mono-exponential probability density function with τ = 1.05 s obtained after applying the Maximum Likelihood Estimation (MLE) method. Source data are provided as a Source Data file.*

Image quality was improved after averaging three frames. Supplementary Figure 13a shows a comparison of ten fluorescent events - corresponding to Cy3B single molecules - with and without averaging. After applying Picasso's Localize[3] module to the original and the averaged video with the same parameters (identification, photon conversion, and fit settings), we found that the localizations' PSF sizes are more uniform (Supplementary Figure 13b). When rendered, the drift-corrected localizations of the averaged video exhibit a more precise look than the original video, as shown in Supplementary Figure 13c. After the 3-frame average, the

mean PSF width, i.e., the Gaussian function's σ of the diffraction-limited spot best fit, was σ = 0.54 µm (calculated as the mean between the σ_x and σ_y).

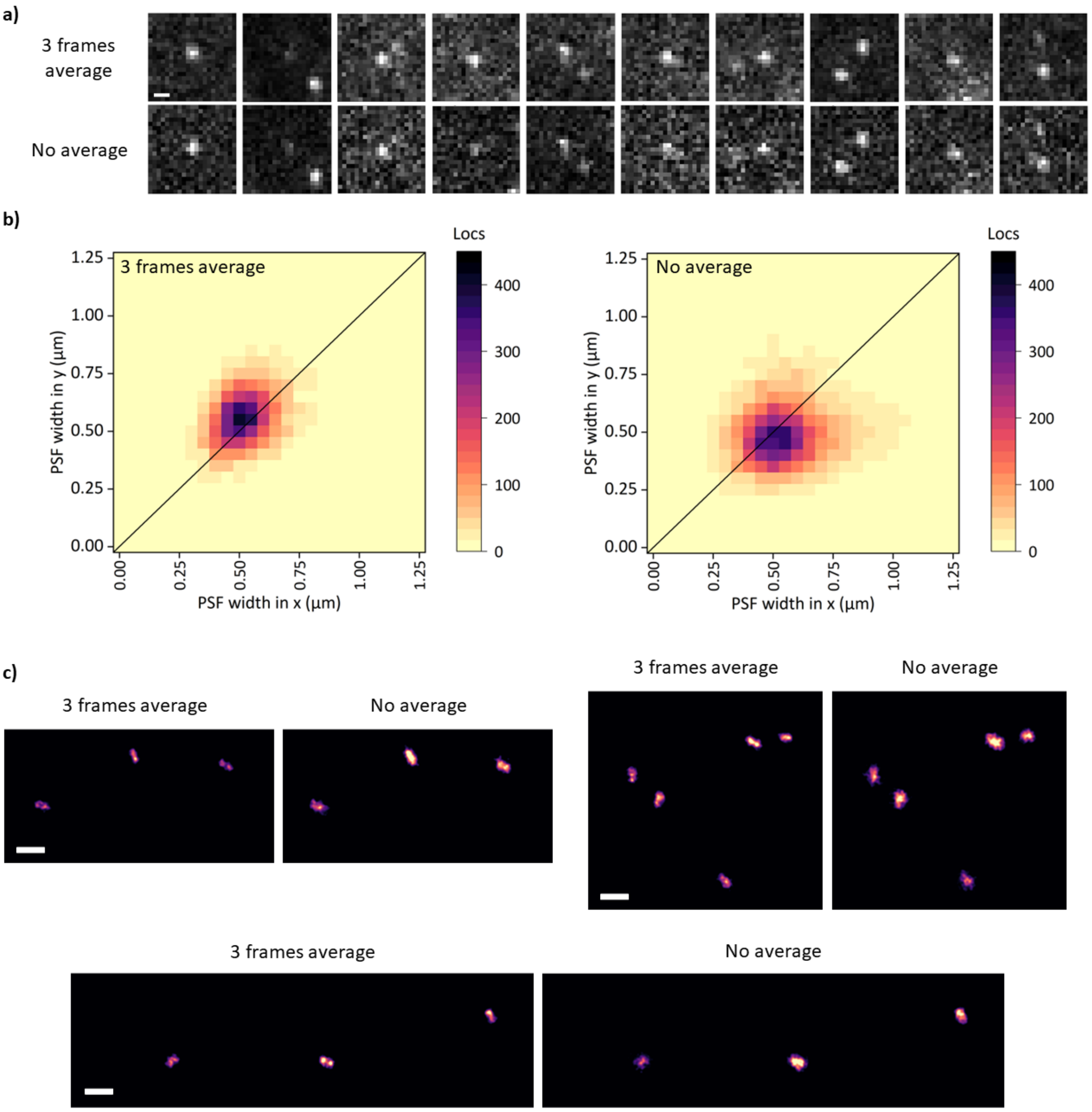

Supplementary Figure 13: Improving image quality by frame averaging with the smartphone-based microscope. *(a) One-to-one comparison of 10 single molecules. (b) PSF width (σ of the Gaussian fit) distribution in x and y. The averaged video exhibits a lower dispersion, meaning the spots are rounder and more similar to each other. (c) Super-resolved images. The same analysis pipeline was applied to both the three-frame-averaged video and the non-averaged (original) video. Nanostructures are sharper when performing the average. Scale bar: 1 µm. Source data are provided as a Source Data file.*

To maximize the number of photons per frame, which in turn improves the localization precision, we tested Cy3B-labeled imagers with two modern oxygen scavenging and triplet quencher systems on the high-end microscope[4,5]: PCA-PCD/Trolox (PPT) and PCA-PCD/COT (PPC). See online Methods for details on their preparation. Supplementary Figure 14 presents distributions of detected photons per frame, signal-to-background ratio (SBR), signal-to-noise ratio (SNR), and radial localization precision. For this analysis, SBR values were obtained from the single-molecule intensity traces using SBR = S/B, where S is the number of photons emitted by the molecule (background corrected) and B is the average background signal per pixel (in photons) obtained as the offset of the fitted 2D Gaussian. PPC buffer presents a higher photon per frame and a higher localization precision[6]. SNR and SBR show no significant differences. PPC buffer was chosen for the DNA-PAINT experiments.

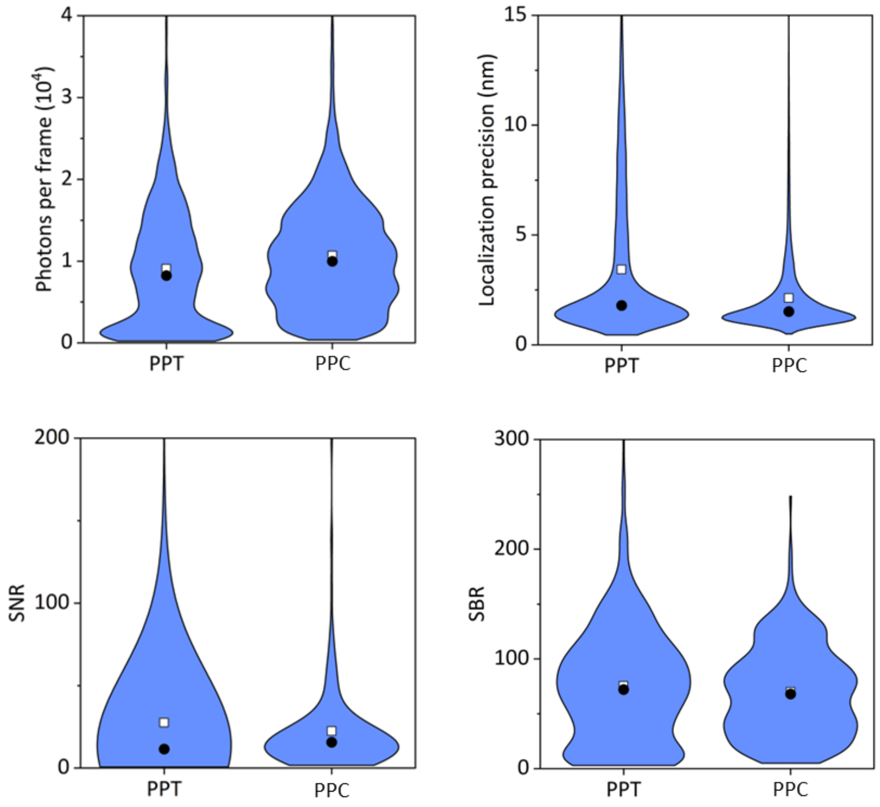

Supplementary Figure 14: Comparison between PPT and PPC imaging buffers. *Distribution of photons per frame, SBR, SNR, and localization precision for 816 single molecules photostabilized with PPT and 556 with PPC. The average (white square) and median (black circle) are shown. Source data are provided as a Source Data file.*

Supplementary Note 12. Example of a super-resolved microtubule network

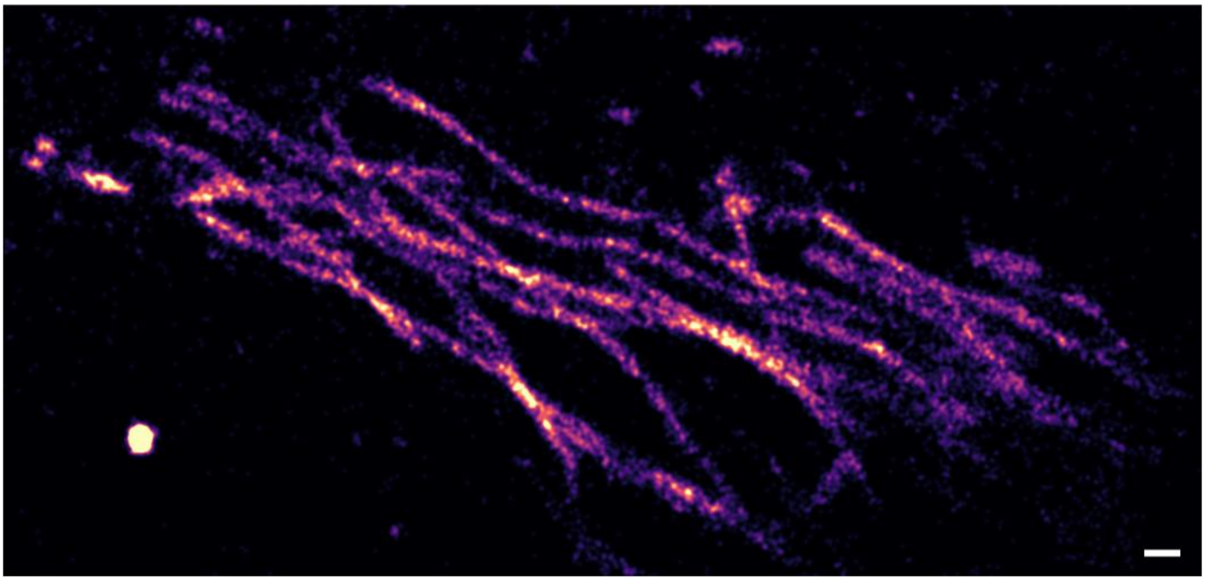

Supplementary Figure 15: Another example of a super-resolved microtubule network. *Super-resolution image (localization density) of a microtubule network of fixed U2OS cells obtained with the smartphone-based microscope. Scale bar: 1 µm.*

Supplementary Note 13. Resolution estimation

To estimate the achieved resolution, we used a parameter-free estimation based on the decorrelation analysis presented by Descloux[7–9]. This method works on images. Therefore, the first step to run the analysis on SMLM data is the rendering of a super-resolved image from the list of localizations. In this work, we chose the fixed Gaussian rendering method. That means each localization is represented by a Gaussian PSF centered on the localized coordinates with the same σ. We set σ to 86 nm for the smartphone-based microscope and σ = 24 nm for the high-end microscope (see Section *Super-resolution benchmark with DNA origami models* in the main text). We ran the analysis on the microtubule imaging data shown in Figure 4 (see main text). The number of localizations was 3.8×10^4 for the smartphone-based setup and 6×10^6 for the high-end microscope. The estimated resolution is approximately 210 nm for the smartphone-based setup and 66 nm for the high-end microscope. The rendering process involves selecting a sub-diffraction pixel size. We tested the influence of the pixel size on the obtained resolution (Supplementary Figure 16).

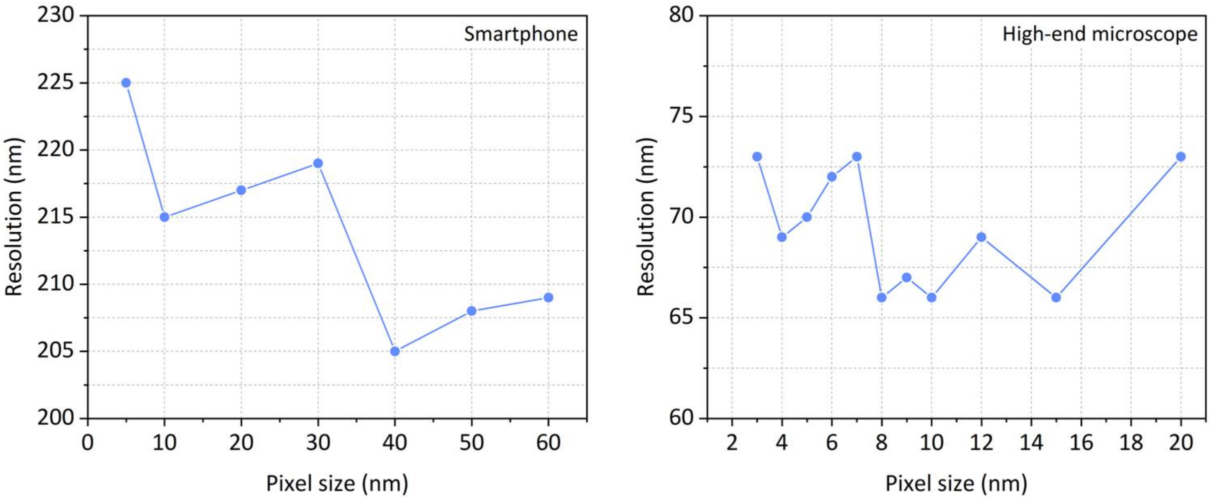

Supplementary Figure 16: Resolution estimation. *Estimated resolution as a function of the pixel size used for rendering the single-molecule localization images. Source data are provided as a Source Data file.*

Supplementary Note 14. TIR excitation design simulations

When focusing on a collimated beam, total internal reflection is successfully achieved when all rays are effectively reflected, as shown in Supplementary Figure 17. Geometrical optics ray tracing simulations were performed using a 532 nm beam at an incident angle θ_i of 80°, a glass substrate (n_i= 1.52), a water medium (n_i= 1.33) and a critical angle θ_c = 61°. We used the Optics Workbench module (https://github.com/chbergmann/OpticsWorkbench) of the open-source software FreeCAD, version 0.20.2 (https://www.freecadweb.org/).

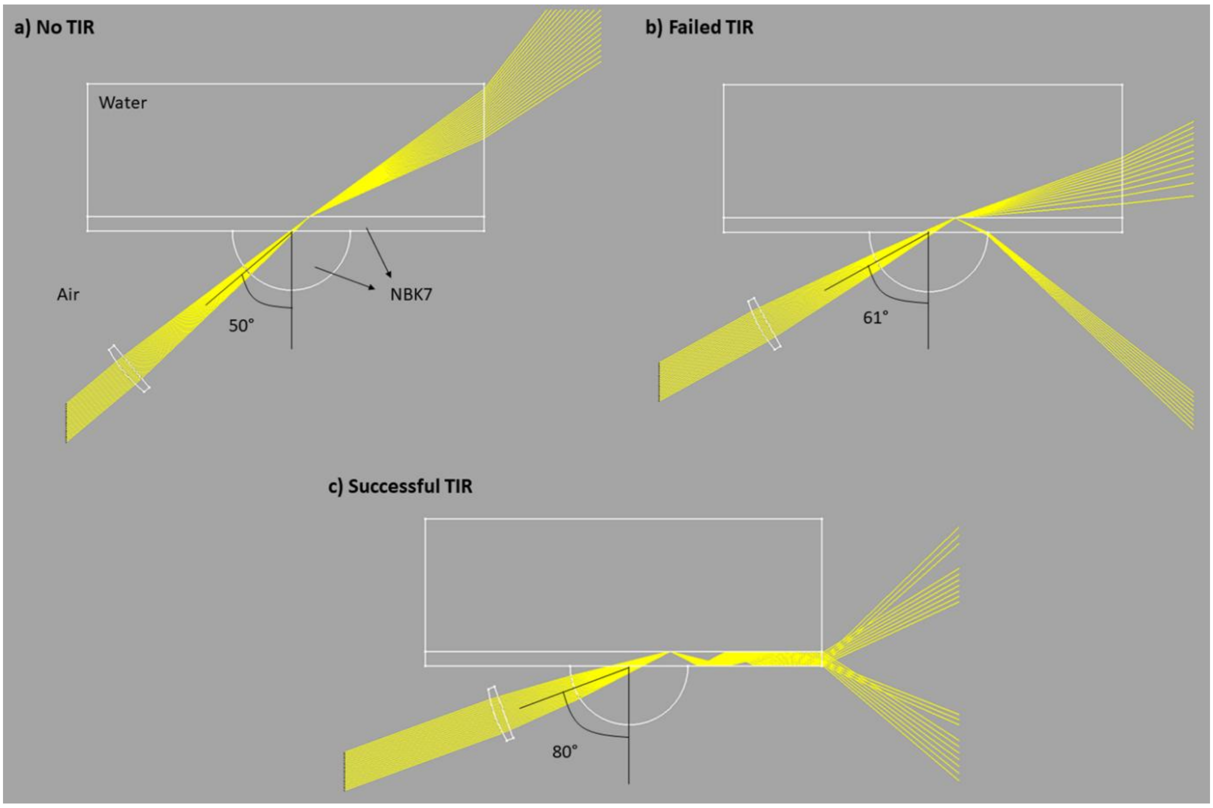

Supplementary Figure 17: Raytracing of the prism-type TIR illumination implemented in the smartphone-based setup. *Different angle of incidence: (a) 50°, (b) 61° and (c) 80°.*

Finally, the penetration depth (d) of the evanescent field can be calculated as[10]:

$$d = \frac{\lambda}{4\pi} \frac{1}{\sqrt{n_i^2 sin^2(\theta_i) - n_s^2}}$$

For our smartphone-based microscope configuration, the penetration depth d is 80 nm.

Supplementary Note 15. Super-resolution on an office desk

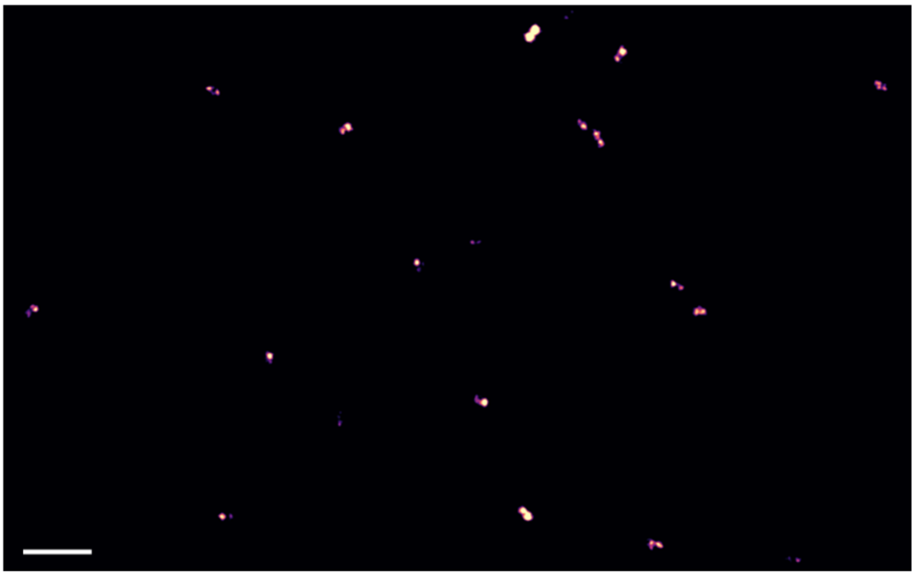

***Supplementary Figure 18**: **DNA-PAINT super-resolved image of several 8HB nanostructures in aqueous solution obtained with the smartphone-based microscope.** The measurement was performed on a standard office desk for ~3.5 h (see Supplementary Note 19). Scale bar: 2 µm.*

Supplementary Note 16. High-end widefield fluorescence microscope

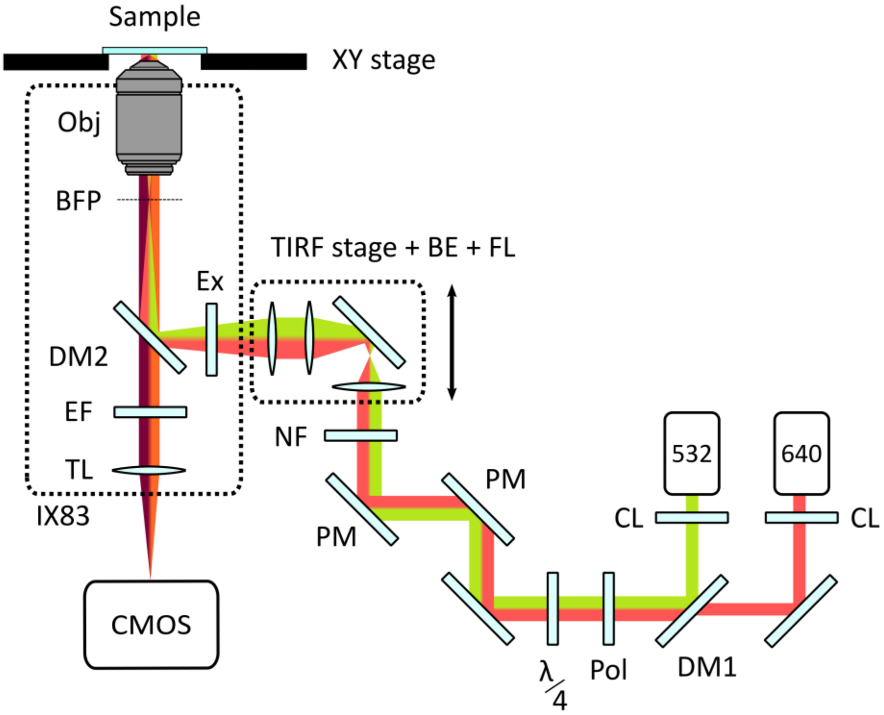

Supplementary Figure 19: Scheme of the High-end Widefield Fluorescence Microscope. *Obj: objective. CL: clean-up filter. DM1/2: dichroic mirror. Pol: polarizer. PM: periscope mirror. NF: neutral density filter. BE: beam expander. FL: focusing lens. Ex: excitation filter. EF: emission filter. BFP: back focal plane. TL: tube lens. CMOS: scientific-grade CMOS camera.*

Supplementary Note 17. 2LS DNA origami staples list

DNA origami nanostructures were designed with cadnanoSQ v0.2.4 (available at https://cadnano.org/legacy.html).

***Supplementary Table 3**: List of staples for the origami core.*

Staple number	Sequence (5' to 3')	Nucleotide length
1	TCCTTTTGATAAGAGCATCAAGAAAACAAA	30
2	AAAACCAAAATAGCGGTGTGATAAATAAGG	30
3	GCTTAGAGCTTAATTGATTACCTGCTTTTTCA	32
4	GAAGTTTTGCCAGAGGTGACCTAATGGCTCAT	32
5	CCATCACCACGTGGCGGCGCTAGGGCACGTAT	32
6	CGCGTACTGGTAATATGAGTAAAAGACCTGAA	32
7	AAAAAAGGACTTTCAAGCCTGTAGGCCACCAC	32
8	CCCCTAAGAGAATATAAAGTATTTTCGAGCCAGTAACCCC	40
9	CCCCTGTGAAATTGTTATCCGCGGGAAGGTGCAAGGCTTGACCGT	45
10	ATTACCATACGGAAATGTTTACCACATACATA	32
11	ATAATAACTAGCAATAGTCAGAGGAATGAAAA	32
12	AAACACTCGAAAGAGGCAGGGAGTATATATTC	32
13	AGAATAGATTTTTTCACATAACCGTAAAGGCC	32
14	AGGCGCATAACTAATGTTAAGAACATTTAATG	32
15	GATTATACGGATTAGAAATTTCATGGTTATAT	32
16	AGTCTCTGAATTTACCGAGCCGCCTCCTCAAG	32
17	CAAAGTTAAGAAAAGTGACGGGAGCATAAAAA	32
18	CGCCTGCATACATTTTACCCTTCTGAGTCTGT	32
19	CCCCTCAATAACCTGTTAACATTATGACCCTGTAATCCCC	40
20	CCCCAACAAAGCTGCTCATTCAGTAAAACGAACTAACCCC	40
21	CCCCCGGAACAACATTACTGCGGAATCGTCATAAATCCCC	40
22	CCCCTCTTTCCAGACGTTAGTAAATCAGCTTGCTTTCCCC	40
23	TGCCTGCACGACGGCCTTCTGGTGTCCAGCCA	32
24	GAGTGTTGTTCCAGTAATCGGAAGGGCAAC	30
25	AAGACTTCAAATATCGCGAGAAAAAGCAAAAG	32
26	CCCCATTCATTGAATCCCCCTCATTTACCCTGACTACCCC	40
27	CCCCAAGCGAAAACCGTCTATCAGGCCCC	29
28	AATTAATGTTATTTCAGTACCAAATAGCTATA	32
29	TAGCAAAAGAGAATCGGACAGTCAAATTCGCG	32

30	CATCAATTCTACTAATTACCAAGTATGCAATG	32
31	TTACTAAAAGGGAGCAATACTTCTTTGATTACCCC	35
32	GCCGGCGACAAATCAAACTCCAACGTCAAAGG	32
33	TAATGAGTGCGCTTAGAGAAGTGGCTTGCA	30
34	GACAAAAGATCGATACCGGAAAACCGGAAC	30
35	TCTGGCCTTGAGCGAGGATCGCACCCGGAAAC	32
36	CCCCATCAGAGCGGGAAGGGAAGACCCC	28
37	CCCCACTTTTGCGGGAGAAGCCTCCGGAGAGGGTAGCCCC	40
38	AATTTAGTCATTCCTTTACGAGGAGGTTT	29
39	AGATTAAACGCTCATTTAGTAAAGGTAA	28
40	CCCCAATCGGCTGTCTTTCCTTAGCAG	27
41	CCCCAAAATCCTGTTTGATGGTCCAGCTGCATTACCCC	38
42	CATGTTCATAGATAAGTCGAGAACTCATTACC	32
43	TACGTTAATGAATAAGCCGGATATACCTGCTC	32
44	AAACTAGCAAAATCTGGATTTAGTCAAAAG	30
45	AAAAGCGTAGATATTTTAAAAGTTTGAGTAACCCC	35
46	ATCAACAAGCTAATGCCATAGTAACGACGATA	32
47	ATTCACTTGCGGCATGTAGAAACCAATCAATCCCC	35
48	TAGCGACATTCATATGTATTCATTGAAACGCA	32
49	CCTAAAACATCTTTGACCGAACTGTACAGACC	32
50	GTAACAGTACCGCCAGGAATAGGGGATTTT	30
51	CCCCCCAAATAAGAAACGATTTTCGGT	27
52	AGACAGCCCGATAGTGGAGCCTTAAACGGG	30
53	CCCCTTTAGCGAACCTCCCGTAAGAACGCGAGGCGTCCCC	40
54	CGTTTTTAGACAGATCTTTAATCCCTGTTT	30
55	ACCACGGAACCGTAAACTAAAGGAGCCGAA	30
56	ATTAAATGTCCTGTAGTCATATGTTAATCGTA	32
57	CAAATAAAATATAAAATACCGAACGAACCACCCCC	35
58	ATAGAAAAGAATCAAGAATCACCACAGAACCG	32
59	TACATGGCCGCGTTTCACCCTCAGAGCCGCC	31
60	CTGTCGTGGGTTCCGATCCACGCTGGTTTGCCCCAGCAGGCGCCCC	46
61	TGCGCTCAAAAAGAATCCGCCTGGCCCTGAGA	32
62	TTTTAGAAATATTCAATGCCTGAGCAGGAAGA	32
63	AAAGGAATTACGAGGAGAACGCGATTGTGA	30
64	AATTATCAAATATCAAAGATTAGACGCGAACT	32
65	CCCCCAGCAGAAGATAAAACAGATTAT	27
66	AGCTCAACTGGGGCGCGAGCTGACAGAGCAT	31

67	TCATAGCCCCCTTATTGCCACCCTGTAGCACC	32
68	CCTACCAATCGTCGCAGTACATAGACTAC	29
69	TCATAATCAAAATCACCGGACCCC	24
70	GAAATACCACAGTGCCCTTTAATGGCCGTCAA	32
71	CGCAAGACAAAGAACGCGTTTTAATGCGGATG	32
72	ACCCATGTATAAGTTACTTGAGCTTGCTCA	30
73	CCCCATTGAGGAAGGTTATCTCAACAGTTGAAAGGACCCC	40
74	GTGCATCTGCAAATATCAGCTCAGCTGATA	30
75	GCTAAACACTCCAAAATGCGCCGAGCAGCGAA	32
76	GCACCCAGAACGAGCGGAGAATAAAATTAACT	32
77	GTACCAGGTAGGATTAGATACAGGAAGCGTCA	32
78	AATCCTTTCAACTAATACCCTCAAATTAACAC	32
79	AATAATAAAAGGAACACACTGAGTCAATAGGA	32
80	TATCATCGATGAGGAAGAGGGTAGTTAAACAG	32
81	GCTTTTGCTAAGGGAACCCCCAGCGGGCTTGA	32
82	CGCGCAGATCATTTCACTGAATATAAGCCCGA	32
83	AGGCTATCATCGGTTACGCAAGGTTTGACCA	31
84	CCAACGCTCTACAATTCGTAGGAAAAGCAAGC	32
85	CCCCTTATTACGCAGTATGTTAGTATC	27
86	AGAAGGATCGGATAAGAGCAAGCCTTCGTCAC	32
87	CCCCCACAAACAAATAAATCCTCATTAAAGCAGGTCATCTGAAAC	45
88	CCCCGAGGACTAAAGACTTTTTCCCTGATAAATTGTCCCC	40
89	CTGAGGTCTGAGAAATCAATATATGTGAGTGCCCC	35
90	CCCCGTCGAAATCCGCGTCATTACCCAAATCAACGTCCCC	40
91	ATTATCACGCCAGCAATTTGCCTTATTTTCGG	32
92	CCACATTCAGGCTGGCAGTAAATTGATTATAC	32
93	GGCAATTCATCATACGTAAAGATAAGTATT	30
94	CCCCATTTATCAAAATCATAGAAGAGTCAATAGTGACCCC	40
95	AAATGGAGGTGAGGCGCCATTACTTTAGG	29
96	CCTGAGTAAGGCCGGAATGAACGGACCCCGGT	32
97	GAACACCCAACATATAAAACCGAGAAAGGTGA	32
98	AATCAGTGAGAATCCTATGCGCCGAACCACCA	32
99	CTTGATACCTCATAGTCTGTATGTGTATCA	30
100	ATCAGGTCAATGCTTTGTCCAATATTACAGGT	32
101	GAGTAATTTTAGTAATGACCATATCTGCGAA	31
102	AAGGTGGCTGAACAAAGCTATCTTGAGCCTAA	32
103	CTATTAAACGGTCACGGAGCTTGAACAGGAAC	32

104	GTATCGGCAGAAAAGCTCAAAAATAATCACCA	32
105	GAGAATCGTCCTTGAATTAGGTTGTTGAATTA	32
106	GAACCTCATCATATTCTCGACAACAACAGTAC	32
107	CCCTCAGAGCCCGTACCTATTATGACGATTGGCCTTGATATTCCCC	46
108	CCACCCTCAGAGCCACTCATCGGCTAGCGTCA	32
109	GCCTTGCTATGGTTGCGATTTTAGCGGGGAAA	32
110	GGAGGCCGCAAATTAAAGAACTCAATCGTCTG	32
111	CCCCGTCACACGACCAGTAAATTGGCAGATTCACCACCCC	40
112	AAGTGTAGGAACGTGGGTTTTTTGGGGTCGAG	32
113	AAAGCTAAAGGTCATCCGTTCTATTTTTTA	30
114	GTCAATCAGGGATCGTACTACGAAATCTCCAA	32
115	GCCAGCCAGCACCGCAGTGCCAATTTTTAT	30
116	TATACCAGGAACGAGTTGACCTTCCGCAGACG	32
117	GGTACGCCAGGCCACCCCAGAACACGCTCATG	32
118	CGAATTATGGCGAGTAGATTTAGATAAAAAT	31
119	TAATATCCTGTCCAGAAACAACGCGCTTAATT	32
120	AGTAATTCCATCCTAAAAGAACGGTATAGAAG	32
121	TGGGTAACTCGAATTCCCACACAACGGGAAAC	32
122	CTATCATACGCACTCATCCTGAACGCTATTTT	32
123	AAACACCATCAGGACGTTGAGATTGTAAAATG	32
124	CCTTAGAATTCTGAATTATACAGTTCGTATTA	32
125	TTTTCATTATGTTTTTCCCAATAATCAAAA	30
126	TGAAGCCTTTACAAAAACGTCAAAGTAATTGA	32
127	CAGTACAACGCCCACGCGTTGAAAGGCACCAA	32
128	GGTTTTTCGCCCTTCAAGCCCGAGATAGGGTT	32
129	TAATCTTGTCATCAGTTGGGAACAGAAAAC	30
130	CCCCACCAGAGCCACCCGTCACCACCCC	28
131	GCTTATCTTGTTTATAAACAGCAAGAAA	28
132	GTGCCGTACCGATTTACTGCGCGTCTACAGGG	32
133	CCTCATTTGTTTTAACGCTGAGACGCCAGCAT	32
134	AACGTGCTGAGTAGACCGTTGTACATTCT	29
135	TCCAATAAATCAATAAACAATAACGGATTC	30
136	AGGCACCGACAATCATATGCGTTATACAAATCCCC	35
137	GGCCAACATAAAACATCGGTCAGTTCAATATC	32
138	CAATGAAAGGAATACCAGAAAATAGCGCCAAA	32
139	GGGAGCCCAAGCACTATTGGAACAAGAGTCCA	32
140	CAACTGTTTCACAATTGTAATCATACGCGCGG	32

141	AGCGTAAGCTATTAGTACGCTGAGACCTTGCT	32
142	GGTGAGAAATGTGTAGAGGCAAGGCATTAACA	32
143	GGTAATAATCAGGGATTGCCGTCGGCGGAGTG	32
144	AATGGGATTTGTTAAATTTAAATTATCTACAA	32
145	TGATAATCCTCAGGAATAACAACCTCACGACG	32
146	CACCCGCCGAGCTAAAGTGAGACCCCTAAA	30
147	ACAACCATACTACAACCAGTTTCAAGAGGGTT	32
148	TTGAATGGAATACGTGAGGAAAAAATATTACC	32
149	GAGTTGCAGGTTTGCGTTTCCAGTCATACGAG	32
150	TGAAAGAGTTTTCATTTATCCTGTACACTA	30
151	CCCCGTAGATGGGCGCATGCGGGCCTCTTCGCTATTCCCC	40
152	TACTAGAACCTCCGGCAACATAGCTAATTTTC	32
153	AGAAAGATACAAGAAGCTTGCCGAGATTTG	30
154	CCAAGTACCCATATTTCGACGACAATCATAAT	32
155	CCCCCCGATTGAGGGACTGGCATGATTAAGACTCCCCCC	39
156	GCAAATGAATGTCAACCAGCTTTAATATTT	30
157	TGGTCAGAAAGAAAGTTATTATTTCAGG	28
158	CCTTTTTTCAGATGAAAATGGAAGAGGAGCGG	32
159	CAAGCGCGGTAATGCCCACCCTCACAATGACA	32
160	TTTGCCAGTAAATCAAAAATCAGAGTATTAAA	32
161	AGAGAATAAGAGCCATATTATTTATCCCAATCCCC	35
162	CCCCGCGATGGCCCACTACGTGAA	24
163	TAAAATACAAACAAAGGAACGAGGATCAAGAG	32
164	CCCCCATTATCATTTTGCGGAACTTGG	27
165	GCTTTCCGTTGCAACGCACAGACCATCAAC	30
166	AAATATTGTAGCAAGGGCAGCACCCCATCTTT	32
167	CCCCCTATTTTTGAGAGGTAAACGTTAATATTTTGTCCCC	40
168	CTTTTACATCGGGAGATAATCCTGCAGATGAT	32
169	CCCCCCGCCACCCTCAGACGATCTAAAGTTTTGTCGCCCC	40
170	GATGGTTTATGCGATTCAGATACATTGCAAAA	32
171	TAGCAGCCTAGCAAGCGATTAGTTAAGAAAAA	32
172	CCCCATGAATCGGCCAGGTCATAGCTGTTTCCTGCCCC	38
173	CCCCATGAAACCGGCGACATTCAACCCC	28
174	CCTTTTTACCAGAAGGAAAGAAACCACAATCA	32
175	CCCCTTATAGTCAGAAGAACTAAAGTACGGTGTCTGCCCC	40
176	CAGGGAAGAGGCTTGCAAAAGAAAATCTTA	30
177	GCGCTAACAAACGTCAAAAGAAGGGAAGGT	30

178	CCGTACTCTTTCGGAATAAACAGTTAATGCCCCCCC	36
179	TACCGAGCGCCAGGGTCGCCATTCCGACGACA	32
180	CCATCACGATTAAAGGTTTGACGAGCGCTGGC	32
181	CCCCAATAACCTTGCTTCTGTAATATC	27
182	ATTGCATCGGATAGCAAACAGTTGAAAAATC	31
183	GAATTAGACGTCACCGTATTTTGTGCAAAGAC	32
184	CCCCTTGAGTTAAGCCCAATAGATAACCCACAAGAACCCC	40
185	CCCCTAAAATTCGCATTAAATTTAGGTCACGTTGGTCCCC	40
186	CCCCCGAGGTGAATTTCCAACGGCTACAGAGGCTTTCCCC	40
187	TAGATAATCAAACAATCTGATTATATTGTTTG	32
188	GACTGTAGTTTTGATGCGGGGTTCATTTGG	30
189	TGACAGGAGGTTGAGGCCAGAATGTGCCTTGA	32
190	GCAAACAATTAAGCAATATTTTAATACAAAAT	32
191	TTTAGACTAAAAAGATTAAGAGGAATGCTGT	31
192	CAGGCAAAATAAAGTGCTAGAGGAGCCAGGGT	32
193	AATATATTTCATCTTCGGGTAATATAGGAATA	32
194	GGTCGCTGCGCATTAAAGCAGATAATTGCG	30
195	CATGTTACTCGGAACGTTTCCATTTAATTGT	31
196	GCGAAAAGGAGCGGAGAAAGGAGCTAAACA	30
197	TTGTAAAAGGTCGACTTAAAGCCTAATTGCGT	32
198	GATATAAGATTAAGAGGGGGTCAGGAAAGCGC	32
199	CCGGAAGCGCGCCATTTTTCCCAGCGTCGGAT	32
200	TCTCCGTGGAACGCCACCCAAAAAAGTCTGGA	32
201	CCCCCAGAAATAAAGAAATTTTATTTGCACGTAAAACCCC	40
202	AGACAGCATTAGCCGTACAACGCTGACGAG	30
203	GATAGCCCGAGATAGAGACGCTCAAACTATCG	32
204	TATAAATCCTGCCCGCTATTGGGCTCCCCGGG	32
205	CCCCTCTTACCAGTATAAAGCCAGACG	27
206	ATCGGTTTATGAATTTTTAGCGTAAACCGCCA	32
207	ACCAATAGGGAACAAATGAGGGGAAGGCTGCG	32
208	AGCTGATTTTTTCACCCTCACATTGGGGTGCC	32
209	AGCACTAAGCCCGAACCCACCAGAGGTTAGAA	32
210	CCCCCCTGCCTAAGGAGGTTTAGTACCCC	29
211	TCAATATGCCCTCATATAAAGCCTAAAGGTGG	32
212	CCCCGTAATAACATCACTTGCCTTTCCTCGTTAGACCCC	39
213	TTGTATAAGCCAGTTCGGCGGAGATTAAGT	30
214	ACAGGTCAACCCTCGTACACCGGAATAAACAA	32

215	CCCCGAAGTTTCATTCCATATAAATTTCGCAAATGGCCCC	40
216	CTTTTTAAAAAGCCTGACAGTAGGCAACATGT	32
217	AGACTTTAACATTTGAAAAGCATCAGCCAGCA	32
218	TTTAACGTAATGGAAACTATTAATGATAGCTT	32
219	GCGCCCAATTTACAGATCTTTCCAACCGAAGC	32
220	AACTATATGTAAATGCCCGGAAGCTTAATTGC	32
221	CCCCACGCCAGCTGGCGAAAGGGGGATGTGCGCGATCGGTCGTAACC	47
222	ACCAGAACCACCACCAGTTCCAGTAGTGTACT	32
223	CGTTAAATAAGAATAATTACCAGAGAGCAACA	32
224	AAGATGATGAAACAAAGTCATTTTTTCGAGCT	32
225	ATTAATTACATTTAACGAGTACCTAAACTCCA	32
226	GTTTGAAATACCGACCAGAGGCTTTAACGCCA	32

***Supplementary Table 4**: List of staples with surface binding function.*

Staple number	Sequence (5' to 3')	Nucleotide length
1	Biotin-TTCCATCACCACGTGGCGGCGCTAGGGCACGTAT	34
2	Biotin-TTATGAAAGTTATAGCCCCCCTCAGACATTCCAC	34
3	Biotin-TTGCCTGATTGCTTTGAAAGTAGTAGCAAAGAAT	34
4	Biotin-TTCGCCTCCCTCAGAGCCAGCGTTTGGTAATCAG	34
5	Biotin-TTATTACCTTAATTTCAAGAACGGTGACCAACTT	34
6	Biotin-TTGGAGAGGCGCAAGCGGAATCGGCAAAATCCCT	34

***Supplementary Table 5**: List of staples with fixed fluorophores.*

Staple number	Sequence (5' to 3')	Nucleotide length
1	TCAAAGCGAACCAGATGATGCAAATCCAATTT-ATTO 542	32
2	TTAGATACCAGTTGATAAATATGCCAAAGCGGTTT-ATTO 647N	35

Supplementary Note 18. 8HB DNA origami staples list

***Supplementary Table 6**: List of staples for the origami core.*

Staple number	Sequence (5' to 3')	Nucleotide length
1	GGGCGAAACAATCAATTGAGGATTGAACGTTA	32
2	ACTATTAGCCAAAGATAGATTTTATCAT	28
3	TTGAGTGTTGAGGGAGATCTAAAAAAGGAGCG	32
4	TTATAAAATTCATTAAATCAACTCAGATG	29
5	TTTGATGGACTTGAGCTCAAACCCGATTGTTT	32
6	CGCTGGTATCACCATCTAAAGGGGTTAG	28
7	CTGGCCCTCCGGAAACTGCCACGCCACGTAAA	32
8	AGACGGGAGCACCGAGGCGGTTTTTCAG	28
9	GGGCGCCATTGCCTTTACGAACCATAACAGTA	32
10	CCAACGCATCGGCAAGCCCTAACGGATT	28
11	AACCTGTCGCGTTTGCAATGGCTATTACAAAA	32
12	GTTGCGCGGAACCAGTAAGAATCAATTA	28
13	GCCTAATGTCAGAGCCCAACAGAGCAAACATC	32
14	AGCCGGAAGAGCCAACACGACAACAATT	28
15	TGTTATCCCCACCACCATGGATTAGAAACAGT	32
16	ATCATGGAGGTTGAGAGTAATGCGAGAG	28
17	GAGGATCCTATTCACATAGAACCCGAGGGGGT	32
18	GCCAAGCAGAATGGGCCTTTAAGCGTCC	28
19	CCCAGTCATTCCAGTACATTATGATTCATTGA	32
20	AGGCGATTACAGGAGAGCATATTCAGAA	28
21	TACGCCAGGGGTCAGTAAGAATTAATCAGGTC	32
22	GAAGGGCAACAGTTTTAACATAGCAAAG	28
23	GCCATTCGCTATTATTAGGTGGCAGGAAGCCC	32
24	ACCGCTTCTGAGACGCTATATAATTCGA	28
25	CAGGAAGAGCGGGGTTATACATTTGCAAACTC	32
26	CAGTTTGGCCGTCGTGCGAACCTTTAAT	28
27	TGTAGATGGGAATAGGTTTCATTCTTTGCGGA	32
28	CGCGTAACCACCACACGGAAGGTTGGAAGGTAGAACTGGC	40
29	GCCGCTACAGGGCGCCAGTTGGCAAAGGTGAAGGAAACG	39
30	AATCAGAGCGGGAGCATGAAAAAGTAGCACCTTACCGAA	39
31	TAAAGGGATTTTAGACTGCAACAGGTCACCAAGAGCAAGA	40
32	AATCCTGAGAAGTGTCAGAGGTGTAATCAGTCCCACAAG	39

33	GCCACCGAGTAAAAGAAATACCGAAGCGTCAGAGGGTAAT	40
34	AGTAATAACATCACTTTATTTTTGCATCTTTTTAACATAA	40
35	TCAAACTATCGGCCTCTGAAAGCGAGCCACCAAAATGAA	39
36	AACAATATTACCGCCAATTCTGGCGCCACCCTCCAAATAA	40
37	AAACGCTCATGGAAACACCAGTCCCACCCTCCAAAATAA	39
38	TCAAATCACCATCAACAATGCCTGGCAGGTCTGATACCG	39
39	TCTAGCTGATAAATTAAAAATTTTAACAAATAGCTTGCTT	40
40	CTATTTTTGAGAGATCGGGAGAAAAAGCGCAAAAAGGAG	39
41	GTCATTGCCTGAGAGTACCAAAAAAGCGTCATTTCACGTT	40
42	GTCAATCATATGTACCGCAAGGCAGCCTTGAGTCAACAGT	40
43	AAAGCCCCAAAAACATAGTAGCAAATGCCCCATTTTCTG	39
44	AAATATTTAAATTGTAAGCTGAAACTGAAACAGTTTTGTC	40
45	TAAAATTCGCATTAACCTGTTTATCCTCAAGACAGCCCT	39
46	GGAATTGACCGCCGCGATAGCCCGAGATAGGG	32
47	TATCTGGTGTACTATGAAATCGGCAAAATCCC	32
48	AGCAGCAATAAACAGGTGCAGCAAGCGGTCCA	32
49	ACACCGCCAGGAACGGGATTGCCCTTCACCGC	32
50	AGATAAAATTTTATAATTTCTTTTCACCAGTG	32
51	CCATTAAAGTCTGTCCAGGCGGTTTGCGTATT	32
52	ACAGACAAGCCTGAGTCGCTTTCCAGTCGGGA	32
53	CTTCTGACTGCTGGTAAACTCACATTAATTGC	32
54	AAAGGGACGCCATTGCGTGTAAAGCCTGGGGT	32
55	GGCAGATTTACCTACATTCCACACAACATACG	32
56	TTTAAATGTATGATATCGAGCTCGAATTCGTA	32
57	CAAGGATAATGCCGGACTGCAGGTCGACTCTA	32
58	TACTTTTGCTACAAAGTAAAACGACGGCCAGT	32
59	TCGGTTGTCTGGAGCAGTAACGCCAGGGTTTT	32
60	TCATACAGCCGGTTGACGGGCCTCTTCGCTAT	32
61	ACTAATAGGGAAGATTGCTGCGCAACTGTTGG	32
62	GGGGCGCGAACGTTAAGGAAACCAGGCAAAGC	32
63	GTCAATAAATTTTTGTCAGCCAGCTTTCCGGC	32
64	ATGGCAATACCGGAATGTCCTGAACAAGAAAA	32
65	AACCTACCACCTAAATCGGCTGTCTTTCCTTA	32
66	GTTTAACGAATCGCAAAAGCAAGCCGTTTTTA	32
67	CCTTTTACATATAACTCATTACCGCGCCCAAT	32
68	TCGCGCAGGTGAATTTGAGGCGTTTTAGCGAA	32
69	AAGAAAACTTTCCCTTCTATTTTGCACCCAGC	32

70	TCATTTGATTGCTTCTAATCTTACCAACGCTA	32
71	GCTTTTGCAGTAAGAGCAGGGAGTTAAAGGCC	32
72	AATACTGCAGATTTAGGAGGGTAGCAACGGCT	32
73	ATCCCCCTACATTATTCTAAAGACTTTTTCAT	32
74	TTTACCCTTCATTATAGCACCAACCTAAAACG	32
75	GAAAGACTTTGAGATGATTATACCAAGCGCGA	32
76	GCTTCAAAGAGAAACAAGATTTGTATCATCGC	32
77	ACTAGAAAAAGCCTGTATCATATTACCCAAAAAATATTGA	40
78	TTAAATAAGAATAAACTCATCAATGGAAACCGATTATCAC	40
79	TTAATTTCATCTTCTGATATCAAATAGCTATCATTACCAT	40
80	AACGCGAGAAAACTTTAAAGAAATAATAATAATGAAACCA	40
81	ATGCTGATGCAAATCCTCAGATGAAGAGATAAAGCGACAG	40
82	CGGCTTAGGTTGGGTTATCGGGAGAAAGTCAGACTGTAGC	40
83	CTGAGAAGAGTCAATAAGGCGAATAGAGAGAACATAATCA	40
84	TGAAAACATAGCGATAAAAGAAGATAACGTCAACCGGAAC	40
85	GTCGCTATTAATTAATAAAATTAAATCCCAATCAGAACCG	40
86	ATGTGAGTGAATAACCATTACCTTGCCAGTTAAGAGCCGC	40
87	AGGAATTACGAGGCATAAAAGAAGAAACAGCTAGACGATT	40
88	ACATTCAACTAATGCAAATGTTTAGTTTATCAAATCCTCA	40
89	AAAGATTCATCAGTTGGGAATCGTAAGGCTCCGTCTCTGA	40
90	ACGAACTAACGGAACACAAATGCTATAATTTTACATGGCT	40
91	GATTTTAAGAACTGGCGACTATTAAACAACTTTAACAGTG	40
92	TCAACTTTAATCATTGATCAAAAAGTAAATGACTGCCTAT	40
93	GAGTAGTAAATTGGGCTCAAATATGATCTAAATGAAAGTA	40
94	TAAGGCTTGCCCTGACGCGAACCATTCCACAGAGAAGGAT	40
95	AGTAATTCTAGAAAATGAGTAACAAGAGCCGT	32
96	ACATGTTCGACTCCTTACCACCAGTATCTTTA	32
97	ATCAACAAAACGGAATCCTGATTAAGTTGAAA	32
98	ATAATATCTACCAGAAATAATCCTTCAATCAA	32
99	AAACCAATTTAAGAAATAATGGAACATCACCT	32
100	TCATTCCAAATAGCAAATTATTTGTGAGAGCC	32
101	GCACTCATTTAAGCCCTGCGTAGACAGTATTA	32
102	TTTTCATCAATATCAGATATACAGCCAGCAGA	32
103	AGCAAGCACCCTGAACAAACAATAAAACATCG	32
104	GGTATTCTAAGCGCATATACCAAGTTAGTCTT	32
105	CCTCCCGAGCCTTTACTATTCATTTACGTGGC	32
106	AAATCAAGTTTTTGTTTGATGAAAATAGAACC	32

107	TACAATTTTATTATTTTTACATTTCAGTAATA	32
108	ACGAGCGTCTTTCACGATCAATAT	24
109	GGTCGCTGCGCCGACACCAAAATAGTGTAGGT	32
110	GCTTTTGCAATTTCTTTTTTGCCATCATATAT	32
111	AGACAGCATTGTATCGGACTGGATTTTCAACG	32
112	ACAGAGGCCCAAAAAACATAAATACCCTGTAA	32
113	GAGGAAGTTGCGAATATTAAACAGAAGCTAAA	32
114	TAATGCCAAGTGAGAAAATCAAAAGCAAAATT	32
115	AAAGAGGCTTTTGCTATAGTCAGACCAATAAA	32
116	TCTTTGACAGACGTTAGATTAAGATCAATTCT	32
117	AACAAAGTAGCGTAACCGCGTTTTTTTCATTT	32
118	CTGATAAACTGTAGCAGACCGGAACGCAAATG	32
119	CCATGTTACTGAGTTTGAGAGTACGAGTAGAT	32
120	GGTCAATCCAAGCCCAAGGTCATTCATATAAC	32
121	TTGAAAGACTCAGAGCTGCTGAATCAACTAAA	32
122	CAACATATATATAAAGATCGCCATATTTAACA	32
123	GCAAACGTGTCCAGGCCAACGCTCAACAG	29
124	ATGATTAAAGCTAATGATATGCGT	24
125	CAATAATTAGATAACATAATT	21
126	AACAAAGTCCATCCTAATAAGGCG	24
127	GCCCTTTCAATAATTTAATGGTTTGAAAT	29
128	AACAATGAAGAACGGGTATTTTAG	24
129	AATTGAGCGAGAACGACAAAG	21
130	TGAGCGCTGTAGGAATATATGTAA	24
131	CTGAACAAATCAGATAACCTC	21
132	AAACAGGGAAGAACGCATCAAAATCATAGGTC	32
133	AATAGCACTTGCGGTAAGACG	21
134	GAAACGATATTAGTTGAGAATCCT	24
135	ACAGCCATATCCTGGTAAATC	21
136	CCCCAGAGCCTAATTTTTTTAATGTTTACATT	32
137	ATAGTTGAGGCTTGCAACACTATCATAAC	29
138	TCGAGGTGGGGATCGTACGCCAAA	24
139	CCTTTAATCGGAACGAATACC	21
140	GAAAATCTTTTGAGGAACAGGTAG	24
141	AAGGAATTTCCATTTAATAAA	21
142	TTCAGCGGCTACGAAGCCAGTCAGGACGTTGG	32
143	TATGGGAAAAAGAACTTATGC	21

144	GTCTTTCCCCCCAGCGGTTTAATT	24
145	CATAGTTACAACGGCCAGAAC	21
146	TACAACGCTTGTGTCGTCAGTGAA	24
147	CGTAACACTTAGCCTTACCCAAATCAACG	29
148	AGGGATAGATAAGGGACAAGAGTA	24
149	TTTTGTCAAACCGTCTTGGCGAGAAAGGAAGG	32
150	TTACCAGCAAGAACGTGCGGGCGC	24
151	CAACCGATTGTTCCAGTCACGCTG	24
152	CGGAAATTTCAAAAGACTTAATGC	24
153	CGTCACCGTGGTTCCGGTTGCTTTGACGAGCA	32
154	CCAGCAAATTGCCCCACTCGTTAG	24
155	TAGCAAGGGAGAGAGTAGGCCGAT	24
156	TCGATAGCCAACAGCTTACGCCAG	24
157	AATCAAGTGGGTGGTTTCAGTGAG	24
158	GCGTTTTCGCGGGGAGATCACGCAAATTAAC	31
159	CCTTATTAGTGCCAGCCTTTGATT	24
160	AAATCACCTCACTGCCAGAAGAAC	24
161	CGCCTCCCAGTGAGCTATATCCAG	24
162	CCACCCTCAGCATAAAAACAGGAA	24
163	CACCAGAAGCTCACAATTTTGACGCTCAATCG	32
164	TTGACAGGTCATAGCTGGAGACAG	24
165	GGCCTTGACCGGGTACTCAACCGT	24
166	TTAAAGCCTTGCATGCGAGGGTAG	24
167	ATTTACCGCGACGTTGGCTATCAG	24
168	TTTGATGATAAGTTGGAACAAGAGAATCGAT	31
169	TTTTAACGCTGGCGAAACTAGCAT	24
170	CCCGTATAGATCGGTGTAATCAGA	24
171	TTCGGAACCCATTCAGGTATAAGC	24
172	TTAAGAGGCTGGTGCCTATTTTGT	24
173	TAGGATTATCGCACTCTAAATCAGCTCATTTT	32
174	GGATAAGTAGGGGACGCCATCAAA	24
175	TATAGCCCGGCGCATCGTAGCCAG	24
176	TTGAAGTACCGCTAAATATG	20
177	CCCGGAACAAACGGCGGA	18
178	CCCACATGTTTCACCCTCAGAACCCCC	27
179	ATAATGCTGTAGCTCACCC	19
180	ACGCCAACATTAAATCCGCAAAGACACCACCC	32

181	CCCGTGTACAGACCAG	16
182	CCGCCACCGGACAGATGAACGCCC	24
183	CCCGCCACCCTCAGAA	16
184	CGTATAACCTCAAATACATTTGGGGATAGCCG	32
185	TGCTGAACGTGCTTTCGCAGGCGAAAATCCTG	32
186	GGATTATATGTGATAAATTTACGAGCATGTAG	32
187	ACCGACCGCTTCTGAAAGTAAGCAAATTAGAG	32
188	CGTTGTAGGAACTGATTTTTCGGTAGAATTAA	32
189	TAATGCGCCAATACTTTGCATTAATGAATCGG	32
190	CGCCTGATTACCTTTTTATAGAAGGCTTATCC	32
191	TGAGAGACTGCTTTGATAGACGGGCATAGCCC	32
192	TCTGGTGAGAAAGGCCGTTTCCTGTGTGAAAT	32
193	AAAGATTCAAAAGGAAAGAGCCGCAACCATCG	32
194	ACAACGACGATAAAAAATGACAACCGCCAGCA	32
195	CCTCGTTTACCAGTAACATAACCGATATATTC	32
196	GAACGGTAAAGCCTCAGTGTACTGAACAACTA	32
197	AAGCAATAATCGTAAAAGGGGGATGTGCTGCA	32
198	AACGAGAAATCTACGTAAACGGGTAAAATACG	32
199	GAAGAAAATGACCATATAGAAAGGGTAATAAG	32

Supplementary Table 7*: List of staples with DNA-PAINT binding strands for the "normal" 8HB origami.*

**Staple number**	**Sequence (5' to 3')**	**Nucleotide length**
1	CCCGGAAAGCCTACAAACAATT TTATCTCCTATACAACTTCC	22+20
2	TTAGACTTGGCGAACGATCAAGAGCTTGACGG TTATCTCCTATACAACTTCC	32+20
3	CCCCGACAACTCGTATGTAATTTAGGC TTATCTCCTATACAACTTCC	28+20
4	CCCCGGAATAAGTTTA TTATCTCCTATACAACTTCC	16+20
5	TATACAAATTCTTACCACAAAGAAATTACGCAGCGACATT TTATCTCCTATACAACTTCC	40+20
6	TTTGCGGAAGTATAAAACGACGACAATAAACA TTATCTCCTATACAACTTCC	32+20
7	GGAGCACTTGTAGCGGTTTGGAACAAGAGTCC TTATCTCCTATACAACTTCC	32+20
8	TAGGGCGCTGGCAAGAACAACTAACAAAAGGGTATGTTA TTATCTCCTATACAACTTCC	39+20
9	GAAGAAAGAATACATTAGAAAATTAAAGGTGG TTATCTCCTATACAACTTCC	32+20
10	CAATAGATCGAAAGGAGGACTCCAACGTCAAA TTATCTCCTATACAACTTCC	32+20
11	TTAATTTTAATTGAGATACCGACAAAAGGTAA TTATCTCCTATACAACTTCC	32+20
12	TAGGGCTTAAAAGTTTACATACATCATATGGT TTATCTCCTATACAACTTCC	32+20
13	ATCTTGACAAGAACCGTTGATAAGATAGGAACGATATAAG TTATCTCCTATACAACTTCC	40+20

14	GCGCATAGGCTGGCTGAGCTTAATCACCACCCCGTACTCA TTATCTCCTATACAACTTCC	40+20
15	TGCTCCTTGATATTCAGGAACGAGGCGCAGAC TTATCTCCTATACAACTTCC	32+20
16	AGTTGATTGCCTTCCTGTAACCGTGCATCTGC TTATCTCCTATACAACTTCC	32+20
17	GTACGGTGATGTGAGCGGATAGGTCACGTTGG TTATCTCCTATACAACTTCC	32+20
18	AATAATTCGCGTCTGCCCAATTCAGAGGGTTCCATGTAC TTATCTCCTATACAACTTCC	39+20
19	CTTTCATCAACATTAATCTGGAAGTGTATCACTCATTTTC TTATCTCCTATACAACTTCC	39+20
20	TTAACCAAACCATTAGTTGCTCAGGTACAAAC TTATCTCCTATACAACTTCC	32+20
21	TTAGTTTGTAGGAACGACGACAGTATCGGCCT TTATCTCCTATACAACTTCC	32+20
22	CAACAGGTCTGCTCATAAATCCGCGACCTGCT TTATCTCCTATACAACTTCC	32+20
23	TAACAAAGCAGGATTACGTCACCATACCAGGC TTATCTCCTATACAACTTCC	32+20

Supplementary Table 8*: List of replacement staples to fold a B-type 8HB origami from the "normal" 8HB origami. The staple number corresponds to the staple number - to be replaced - of Supplementary Table 7. The protruding strands that are complementary to the Ebola virus RNA fragment (in orange) go from staple number 14 to 24. The strands that correspond to the control site go from staple 4 to 13 (in blue).*

**Staple number**	**Sequence (5' to 3')**	**Nucleotide length**
4	CCCCGGAATAAGTTTA TTATCTCCTATACAACTTCC	16+20
5	TATACAAATTCTTACCACAAAGAAATTACGCAGCGACATT TTATCTCCTATACAACTTCC	40+20
6	TTTGCGGAAGTATAAAACGACGACAATAAACA TTATCTCCTATACAACTTCC	32+20
7	GGAGCACTTGTAGCGGTTTGGAACAAGAGTCC TTATCTCCTATACAACTTCC	32+20
8	TAGGGCGCTGGCAAGAACAACTAACAAAAGGGTATGTTA TTATCTCCTATACAACTTCC	39+20
9	GAAGAAAGAATACATTAGAAAATTAAAGGTGG TTATCTCCTATACAACTTCC	32+20
10	CAATAGATCGAAAGGAGGACTCCAACGTCAAA TTATCTCCTATACAACTTCC	32+20
11	TTAATTTTAATTGAGATACCGACAAAAGGTAA TTATCTCCTATACAACTTCC	32+20
12	TAGGGCTTAAAAGTTTACATACATCATATGGT TTATCTCCTATACAACTTCC	32+20
13	TTAACCAAACCATTAGTTGCTCAGGTACAAAC TTATCTCCTATACAACTTCC	32+20
14	ATCTTGACAAGAACCGTTGATAAGATAGGAACGATATAAG CTCACAAAGCTGTTG	40+15
15	GCGCATAGGCTGGCTGAGCTTAATCACCACCCCGTACTCA CTCACAAAGCTGTTG	40+15
16	TGCTCCTTGATATTCAGGAACGAGGCGCAGAC CTCACAAAGCTGTTG	32+15
17	AGTTGATTGCCTTCCTGTAACCGTGCATCTGC CTCACAAAGCTGTTG	32+15
18	GTACGGTGATGTGAGCGGATAGGTCACGTTGG CTCACAAAGCTGTTG	32+15
19	AATAATTCGCGTCTGCCCAATTCAGAGGGTTCCATGTAC CTCACAAAGCTGTTG	39+15
20	CTTTCATCAACATTAATCTGGAAGTGTATCACTCATTTTC CTCACAAAGCTGTTG	40+15

21	TTAACCAAACCATTAGTTGCTCAGGTACAAAC CTCACAAAGCTGTTG	32+15
22	TTAGTTTGTAGGAACGACGACAGTATCGGCCT CTCACAAAGCTGTTG	32+15
23	CAACAGGTCTGCTCATAAATCCGCGACCTGCT CTCACAAAGCTGTTG	32+15
24	TAACAAAGCAGGATTACGTCACCATACCAGGC CTCACAAAGCTGTTG	32+15

Supplementary Table 9*: List of replacement staples to connect an A-type 8HB origami with a B-type 8HB origami. The strands that are complementary to each other have the same number in their label, e.g. A1 hybridized with B1. The protruding sequence is colored in orange.*

Staple number	Staple label	Sequence (5’ to 3’)	Nucleotide length
1 (Suppl. Table 12)	A1	ACCCGTCGGATTCTCCGTG TCAATCTCACC	19+11
179 (Suppl. Table 6)	A2	ATAATGCTGTAGCTCA AACCTGAAAAT	16+11
182 (Suppl. Table 6)	A3	CCGCCACCGGACAGATGAACG CATCATGCGTA	21+11
178 (Suppl. Table 6)	A4	CCCACATGTTTCACCCTCAGAACC TGTACTTACGG	24+11
2 (Suppl. Table 7)	B1	TTAGACTTGGCGAACGATCAAGAGCTTGACGG GGTGAGATTGA	32+11
1 (Suppl. Table 7)	B2	CCCGGAAAGCCTACAAACAATT ATTTTCAGGTT	22+11
3 (Suppl. Table 7)	B3	CCCCGACAACTCGTATGTAATTTAGGC TACGCATGATG	27+11
180 (Suppl. Table 6)	B4	ACGCCAACATTAAATCCGCAAAGACACCA CCGTAAGTACA	29+11

Supplementary Table 10*: Sequence of the imager and bridge strand. Ebola virus (EBOV) RNA fragment.*

Strand type	Sequence (5’ to 3’)	Nucleotide length
Imager	BHQ2-AAGTTGTAATGAAGA-Cy3B	15
Bridge	TGGCGTCATCTCCAG TTATCTCCTATACAACTTCC	15+20
EBOV RNA	CUGGAGAUGACGCCA CAACAGCUUUGUGAG	15+15

Supplementary Table 11*: List of staples with surface binding function.*

Staple number	Sequence (5’ to 3’)	Nucleotide length
1	Biotin-GAATTATCTTAGTATCCAGAACGCGCCTGTTT	32
2	Biotin-ACAGAAATTTCAAATATATTAAACCAAGTACC	32
3	Biotin-CCTGAGCAGCTTAGATGAGGTTTTGAAGCCTT	32
4	Biotin-AATAGTAAGATACATACACCCTCAGCAGCGAA	32
5	Biotin-CGGATTGCTGAATTACTACACTAAAACACTCA	32

6	Biotin-TGGCTTAGACCTTCATACCGAACTGACCAACT	32

***Supplementary Table 12**: List of staples with fixed fluorophores.*

Staple number	Sequence (5’ to 3’)	Nucleotide length
1	GGAGGTTTCCGTAATGGAGTAACAACCCGTCGGATTCTCCGTGCCC-ATTO 542	46
2	ATTO 542-CCCAGAGGCATTTTCGAGCCAGTAATAAGAGAAAAAGAAACTTTGCCCTAGAAGTA	56

Supplementary Note 19. Experimental parameters

Supplementary Table 13 reports on the relevant experimental parameters used during the acquisition of images and videos. Supplementary Table 14 reports on the data type and the associated file properties and pre-processing of the recordings performed with the smartphones.

The dead time between frames for the Samsung Galaxy S22 Ultra was determined by computing the ratio between total measurement time and the number of frames for a long measurement of 14,554 frames performed under the same conditions reported in the Methods section, particularly, raw acquisition mode and 40% sensor cropping. The ratio corresponds to the total time per frame (frame time), which was determined to be 250.1 ms when we used an exposure time of 250 ms. Thus, the influence of the dead time between frames in the total measurement time of our experiments was negligible, meaning that the frame time can be considered to be the same as the exposure time. For instance, the total measurement time of the image in Figure 2c was 4.0016 seconds, instead of 4 seconds.

Supplementary Table 13*: Imaging parameters of recorded videos. The ISO parameter is reported for smartphone cameras. The irradiance is reported as the equivalent top-hat excitation using the reported waist (see Methods) and the measured power at the sample plane.*

Dataset	λ_{exc} and Irradiance (kW/cm^2)	Exp. time (ms)	Nr. of frames	Sample, DNA origami, and fluorophore	Buffer	ISO	Setup	Comments
Figure 2b	640 nm<br>0.68	100	10	2LS with the fixed ATTO 647N	PPC	-	High-end microscope	The image is an average of 10 frames. Total measurement time 1s.
Figure 2c	532 nm<br>0.76	250	16	2LS with the fixed ATTO 542	PPC	1000	Smartphone-based microscope with Samsung Galaxy S22 Ultra	The image shown is an average of 16 frames. Total measurement time 4 s.
Figure 2d	532 nm<br>0.76	250	234	2LS with the fixed ATTO 542	PPC	1000	Smartphone-based microscope with Samsung Galaxy S22 Ultra	Single-molecule intensity traces. No averaging.
Figure 3b	532 nm<br>0.76	250	40,785	8HR monomer with Cy3B fluorogenic imager	PPC	1000	Smartphone-based microscope with Samsung Galaxy S22 Ultra	Super-resolved image from a 3-frame average DNA-PAINT video. Effective exposure time 750 ms.

Figure 3c	532 nm 3.2	100	18,000	8HR monomer with Cy3B fluorogenic imager	PPC	-	High-end microscope	Super-resolved image from DNA-PAINT video.
Figure 4b	532 nm 0.76	200	28,245	Microtubules (U2OS cell) targeted with Cy3B imager	PPC	640	Smartphone-based microscope with Samsung Galaxy S22 Ultra	Super-resolved image from a 3-frame average DNA-PAINT video. Effective exposure time 600 ms.
Figure 4d	532 nm 1.9	50	36,000	Microtubules (U2OS cell) targeted with Cy3B imager	1xPBS	-	High-end microscope	Super-resolved image from DNA-PAINT video.
Figure 5c "Control"	532 nm 0.56	100	18,000	8HR monomer with Cy3B fluorogenic imager	PPC	-	High-end microscope	Super-resolved image from DNA-PAINT video. No target RNA, no bridge added.
Figure 5c "Detected"	532 nm 0.56	100	3,331	8HR monomer with Cy3B fluorogenic imager	PPC	-	High-end microscope	Super-resolved image from DNA-PAINT video. The target RNA and the bridge strand were incubated.
Figure 5d "Control"	532 nm 0.76	200	8,586	8HR monomer with Cy3B fluorogenic imager	PPC	800	Smartphone-based microscope with Samsung Galaxy S22 Ultra	Super-resolved image from a 5-frame average DNA-PAINT video. Effective exposure time 1 s. No target RNA, no bridge added.
Figure 5d "Detected"	532 nm .76	250	5,006	8HR monomer with Cy3B fluorogenic imager	PPC	1000	Smartphone-based microscope with Samsung Galaxy S22 Ultra	Super-resolved image from a 4-frame average DNA-PAINT video. Effective exposure time 1 s. The target RNA and the bridge strand were incubated.
Suppl. Figure 5	640 nm 0.44	100	-	2LS with the fixed ATTO 647N	PPC	-	High-end microscope	Intensity traces of Figure 2b.
Suppl. Figure 7a	532 nm 0.76	250	-	2LS with the fixed ATTO 542	PPC	600	Smartphone-based microscope with iPhone 14 Pro	The image is an average of 16 frames.
Suppl. Figure 7b	640 nm 1.8	100	-	2LS with the fixed ATTO 647N	PPC	-	High-end microscope	The image is an average of 10 frames.
Suppl. Figure 8a	532 nm 0.76	80	-	2LS with the fixed ATTO 542	PPC	2850	Smartphone-based microscope with Huawei P20 Pro	The image is an average of 48 frames.
Suppl. Figure 8b	640 nm 0.68	100	-	2LS with the fixed ATTO 647N	PPC	-	High-end microscope	The image is an average of 10 frames.

Suppl. Figure 9	532 nm 0.87	100	1,026 & 618	8HR monomer with the fixed ATTO 542	PPC	-	High-end microscope	SBR and SNR statistics of single ATTO 542 molecules retrieved from two videos. The SBR was obtained from a 12-frame averaged image.
Suppl. Figure 13	532 nm 0.76	250	40,785	8HR monomer with Cy3B fluorogenic imager	PPC	1000	Smartphone-based microscope with Samsung Galaxy S22 Ultra	Super-resolved image from a 3-frame average DNA-PAINT video. Effective exposure time 750 ms.
Suppl. Figure 14	532 nm 3.2	100	18,000	8HR monomer with Cy3B fluorogenic imager	PPT and PPC	-	High-end microscope	Super-resolved image from DNA-PAINT video. Buffer performance experiment.
Suppl. Figure 15	532 nm 0.76	200	25,725	Microtubules (U2OS cell) targeted with Cy3B imager	PPC	640	Smartphone-based microscope with Samsung Galaxy S22 Ultra	Super-resolved image from a 3-frame average DNA-PAINT video. Effective exposure time 600 ms.
Suppl. Figure 18	532 nm 0.76	250	51,099	8HR monomer with Cy3B fluorogenic imager	PPC	1000	Smartphone-based microscope with Samsung Galaxy S22 Ultra	Super-resolved image from a 3-frame average DNA-PAINT video. Effective exposure time 750 ms.

Supplementary Table 14*: File sizes, format and pre-processing steps for the smartphones used in this work.*

Type of measurement	Dataset of	Nr. of frames	File size	Format	Pre-processing	Smartphone
Single-molecule intensity trace	Figure 2c	331	~1 GB (3.05 MB per frame)	MCRAW, uncompressed	Converted to 16-bit DNG	Samsung Galaxy S22 Ultra
DNA-PAINT	Figure 3b	40,785	~121 GB (3.05 MB per frame)	MCRAW, uncompressed	Converted to 16-bit DNG	Samsung Galaxy S22 Ultra
DNA-PAINT	Figure 4b	28,245	~84 GB (3.05 MB per frame)	MCRAW, uncompressed	Converted to 16-bit DNG	Samsung Galaxy S22 Ultra
DNA-PAINT	Figure 5d "Control"	8,586	~25 GB (3.05 MB per frame)	MCRAW, uncompressed	Converted to 16-bit DNG	Samsung Galaxy S22 Ultra
DNA-PAINT	Figure 5d "Detected"	5,006	~15 GB (3.05 MB per frame)	MCRAW, uncompressed	Converted to 16-bit DNG	Samsung Galaxy S22 Ultra
Single-molecule intensity trace	Suppl. Figure 7a	152	129 MB (~0.85 MB per frame)	MOV, uncompressed (ProRes encoding)	Converted to 32-bit TIFF	iPhone 14 Pro

Single-molecule intensity trace	Suppl. Figure 8a	523	5.23 MB (~9 kB per frame)	8-bit MP4, compressed (H.264 encoding)	Converted to 8-bit TIFF	Huawei P20 Pro
DNA-PAINT	Suppl. Figure 13	40,785	~124 GB (3.05 MB per frame)	MCRAW, uncompressed	Converted to 16-bit DNG	Samsung Galaxy S22 Ultra
DNA-PAINT	Suppl. Figure 15	25,725	~78 GB (3.05 MB per frame)	MCRAW, uncompressed	Converted to 16-bit DNG	Samsung Galaxy S22 Ultra
DNA-PAINT	Suppl. Figure 18	51,099	~156 GB (3.05 MB per frame)	MCRAW, uncompressed	Converted to 16-bit DNG	Samsung Galaxy S22 Ultra

Supplementary References

1. Strauss, M. T., Schueder, F., Haas, D., Nickels, P. C. & Jungmann, R. Quantifying absolute addressability in DNA origami with molecular resolution. *Nat. Commun.* **9**, 1–7 (2018).
2. Endesfelder, U., Malkusch, S., Fricke, F. & Heilemann, M. A simple method to estimate the average localization precision of a single-molecule localization microscopy experiment. *Histochem. Cell Biol.* **141**, 629–638 (2014).
3. Schnitzbauer, J., Strauss, M. T., Schlichthaerle, T., Schueder, F. & Jungmann, R. Super-resolution microscopy with DNA-PAINT. *Nat. Protoc.* **12**, 1198–1228 (2017).
4. Aitken, C. E., Marshall, R. A. & Puglisi, J. D. An oxygen scavenging system for improvement of dye stability in single-molecule fluorescence experiments. *Biophys. J.* **94**, 1826–1835 (2008).
5. Olivier, N., Keller, D., Gönczy, P. & Manley, S. Resolution Doubling in 3D-STORM Imaging through Improved Buffers. *PLoS One* **8**, 1–9 (2013).
6. Thompson, R. E., Larson, D. R. & Webb, W. W. Precise nanometer localization analysis for individual fluorescent probes. *Biophys. J.* **82**, 2775–2783 (2002).
7. Descloux, A., Grußmayer, K. S. & Radenovic, A. Parameter-free image resolution estimation based on decorrelation analysis. *Nat. Methods* **16**, 918–924 (2019).
8. Dance, A., Descloux, A., Grußmayer, K. S. & Radenovic, A. Amendments Publisher Correction : Molecular motion on ice Addendum : Parameter-free image resolution estimation based on decorrelation analysis. *Nat. Methods* **17**, 1061–1063 (2020).
9. Descloux, A. C., Grußmayer, K. S. & Radenovic, A. Parameter-free rendering of single-molecule localization microscopy data for parameter-free resolution estimation. *Commun. Biol.* **4**, 1–6 (2021).
10. Szalai, A. M. *et al.* Three-dimensional total-internal reflection fluorescence nanoscopy with nanometric axial resolution by photometric localization of single molecules. *Nat. Commun.* **12**, 1–15 (2021).
